# Automatic, But Not Autonomous: Implicit Adaptation Is Modulated by Goal-Directed Attentional Demands

**DOI:** 10.1523/ENEURO.0243-25.2026

**Published:** 2026-03-13

**Authors:** Joshua Liddy, Sean R. O’Bryan, Alexander Daskalopoulos, Joo-Hyun Song

**Affiliations:** ^1^Department of Kinesiology, University of Massachusetts Amherst, Amherst, Massachusetts 01003; ^2^Department of Cognitive and Psychological Sciences, Brown University, Providence, Rhode Island 02906; ^3^Carney Institute for Brain Science, Brown University, Providence, Rhode Island 02906

**Keywords:** dual task, goal-directed attention, implicit adaptation, motor learning, sensory prediction error, state-space model

## Abstract

Implicit adaptation recalibrates movements based on sensory prediction errors. It is often characterized as automatic and resource-independent, suggesting that it is insulated from cognitive influence. Here, we asked whether implicit adaptation is sensitive to goal-directed attentional demands imposed by a concurrent visual task. Across two experiments, we used clamped visual feedback to measure implicit adaptation while human adults (49 females, 23 males) monitored a rapidly changing visual stream for targets. In Experiment 1, participants performing the visual task showed modest early enhancement in implicit adaptation relative to a single-task control condition. In Experiment 2, adding response-contingent feedback to the visual task led to stronger and more sustained enhancement. Visual task accuracy and implicit adaptation were uncorrelated, arguing against resource competition. Model-based analyses revealed elevated error sensitivity under dual-task conditions, with individual differences reflecting an inverse relationship between error sensitivity and retention. These patterns are compatible with arousal-mediated modulation of cerebellar error processing and hierarchical models of cerebellar learning. Together, these findings suggest that implicit adaptation is automatic but not autonomous: while it operates outside voluntary control, it appears open to the physiological states in which errors are experienced.

## Significance Statement

Implicit adaptation helps us fine-tune movements using sensory feedback, keeping our actions calibrated as our body or the environment changes. This learning process operates automatically, meaning we cannot control it even if we try. However, does that mean it is completely independent of what else we are doing? We found that when people performed a visual monitoring task at the same time as a reaching task, they adapted more, not less. This enhancement was strongest when the visual task included feedback that sustained engagement. These findings suggest that automatic learning is shaped not only by the errors we experience but also by the context in which they occur.

## Introduction

Skilled behaviors, such as riding a bike over uneven terrain, rely on learning contingencies between sensory states and actions—such as how handlebar movements alter heading direction. Once such contingencies are learned, behaviors become more automatic and less effortful (Logan, 2018). For example, people are often unaware of countersteering on a bike, where a slight turn of the handlebars in the opposite direction precedes leaning into a turn.

But what happens when these contingencies are altered, such as riding a bike with reversed handlebars (Magnard et al., 2024)? Implicit adaptation, a process that underlies the ability to adjust actions to internal or external changes, supports this recalibration (Shadmehr et al., 2010; Krakauer et al., 2019). This process relies on sensory prediction errors (SPEs)—discrepancies between the predicted and observed sensory consequences of movements—to update actions (Mazzoni and Krakauer, 2006; Tseng et al., 2007).

Implicit adaptation is an automatic process, operating in parallel with explicit strategies (Taylor and Ivry, 2011; Taylor et al., 2014). Its automaticity is reflected in several key features. First, it persists even when explicit strategies cancel target error, leading to worse performance (Mazzoni and Krakauer, 2006; Taylor and Ivry, 2011). Second, it responds robustly to noncontingent feedback, even when people are instructed to ignore it (Morehead et al., 2017; Kim et al., 2018). Third, it depends on precise feedback timing—delayed feedback disrupts implicit adaptation but spares explicit strategies (Brudner et al., 2016; Schween and Hegele, 2017). Finally, it produces aftereffects, indicating that it cannot be immediately reversed, unlike explicit strategies, which can be flexibly turned on and off (Mazzoni and Krakauer, 2006; McDougle et al., 2015).

A common feature of automatic processes is their presumed independence from cognitive resources (Shiffrin and Schneider, 1984; Logan, 2018). However, whether implicit adaptation operates independently of resource-limited cognitive systems, like attention, remains under debate. Early studies showed that adaptation was impaired by secondary task demands, suggesting that it is sensitive to attentional demands (Redding et al., 1985; Redding and Wallace, 1985). More recent studies similarly report that attentional demands disrupt adaptation (Eversheim and Bock, 2001; Taylor and Thoroughman, 2007, 2008; Galea et al., 2010; Scheerer et al., 2016; Wang et al., 2022; Tsay et al., 2024b). Yet interpreting these findings is complicated by the operation of multiple learning processes (Taylor et al., 2014; Bond and Taylor, 2015; McDougle et al., 2015).

Given that multiple learning processes are often engaged, it is important to clarify which components are influenced by attentional demands and in what ways. One possibility is that attentional demands interfere with explicit strategies, which depend on resource-limited systems like spatial working memory (Anguera et al., 2010; Christou et al., 2016; O’Bryan et al., 2025). Alternatively, implicit adaptation itself may be sensitive to attentional demands (Scheerer et al., 2016; Tsay et al., 2024b). For instance, task-irrelevant visual distractors can attenuate implicit adaptation, suggesting that this process may be susceptible to attentional competition (Tsay et al., 2024b).

To further investigate how implicit adaptation is influenced by attentional demands, we used clamped visual feedback to isolate it (Morehead et al., 2017), while human adults simultaneously performed a rapid serial visual presentation (RSVP) task. In this reaching task, the feedback cursor is noncontingent: its radial position matches the hand, but its angular position is fixed relative to the target. Participants are told that the feedback does not match their movement direction and should be ignored. Even so, gradual changes in the hand angle reliably emerge, consistent with implicit adaptation driven by SPEs (Morehead et al., 2017; Kim et al., 2018). Unlike past work that examined task-irrelevant visual distractors (Tsay et al., 2024b), we added a competing visual signal that was task-relevant, which could produce attenuation or engage different mechanisms entirely.

In Experiment 1 (Exp 1), we examined whether implicit adaptation was modulated by a concurrent visual task. Participants either ignored the RSVP stream [single task (ST)] or monitored it while reaching and reported target counts after reaching [dual task (DT)]. In Experiment 2 (Exp 2), we introduced response-contingent feedback to sustain engagement with the RSVP task, allowing us to assess whether transient modulation observed in the DT condition reflected sensitivity of implicit adaptation to attentional demands. To complement behavioral measures, we conducted computational modeling to gain insights into how attentional demands alter the learning parameters of implicit adaptation.

## Materials and Methods

### Participants

A total of 79 male and female volunteers recruited from a SONA research pool or by fliers were enrolled and received course credit (*n* = 54) or monetary compensation (*n* = 25; $10/hour) for participating. Participants were right-handed with normal color vision, normal or corrected-to-normal visual acuity, normal hearing, and no orthopedic injuries or neuromuscular disorders impacting arm or hand function. All experimental procedures were approved by the Brown University IRB, and informed consent was obtained from all participants.

Participants were randomly assigned to experimental groups in Exp 1. Exp 2 only contained one group to compare with those from Exp 1. Table 1 contains group demographics. We adopted group sample sizes consistent with prior experiments (Morehead et al., 2017), except for those conducted online (Avraham and Ivry, 2024). See Appendix 1 (https://doi.org/10.5281/zenodo.18510924) for details regarding excluded data (*n* = 7).

**Table 1.** Group demographics

Experiment	Group	*n*	Age (years)	Sex
1	ST	24	19.6 ± 1.3	19 F/5 M
	DT	24	19.8 ± 1.9	15 F/9 M
2	DT feedback (DT_F_)	24	22.5 ± 4.2	15 F/9 M

The group mean and standard deviation are indicated for age. Race and ethnicity were obtained following NSF guidelines: Hispanic (10 or 13.9%), white (24 or 33.3%), Black (3 or 4.2%), American Indian or Alaskan Native (1 or 1.4%), Asian (22 or 30.6%), other (9 or 12.5%), and not reported (3 or 4.2%). We did not control the distribution of sex or race/ethnicity among the groups.

### Equipment

Participants sat in front of an Apple iMac computer with a 21 in monitor (resolution, 1,920 × 1,080 pixels; refresh rate, 60 Hz). The horizontal distance between the screen and the participants’ eyes was ∼57 cm (1 cm ∼1° visual angle). Participants held a stylus in their right hand and performed reaching movements by moving it on 46 × 26 cm digitizing screen (Magic Touch, Keytec) centered horizontally on the right shoulder. Their right arm was blocked from view by an opaque surface, and the lights were turned off to minimize peripheral distractions. Key press responses were recorded with their left hand. MATLAB (R2015a) and Psychtoolbox-3 (Kleiner et al., 2007) were used to present stimuli and record movements at 200 Hz.

### Tasks

#### Reaching task

**Figure 1.**
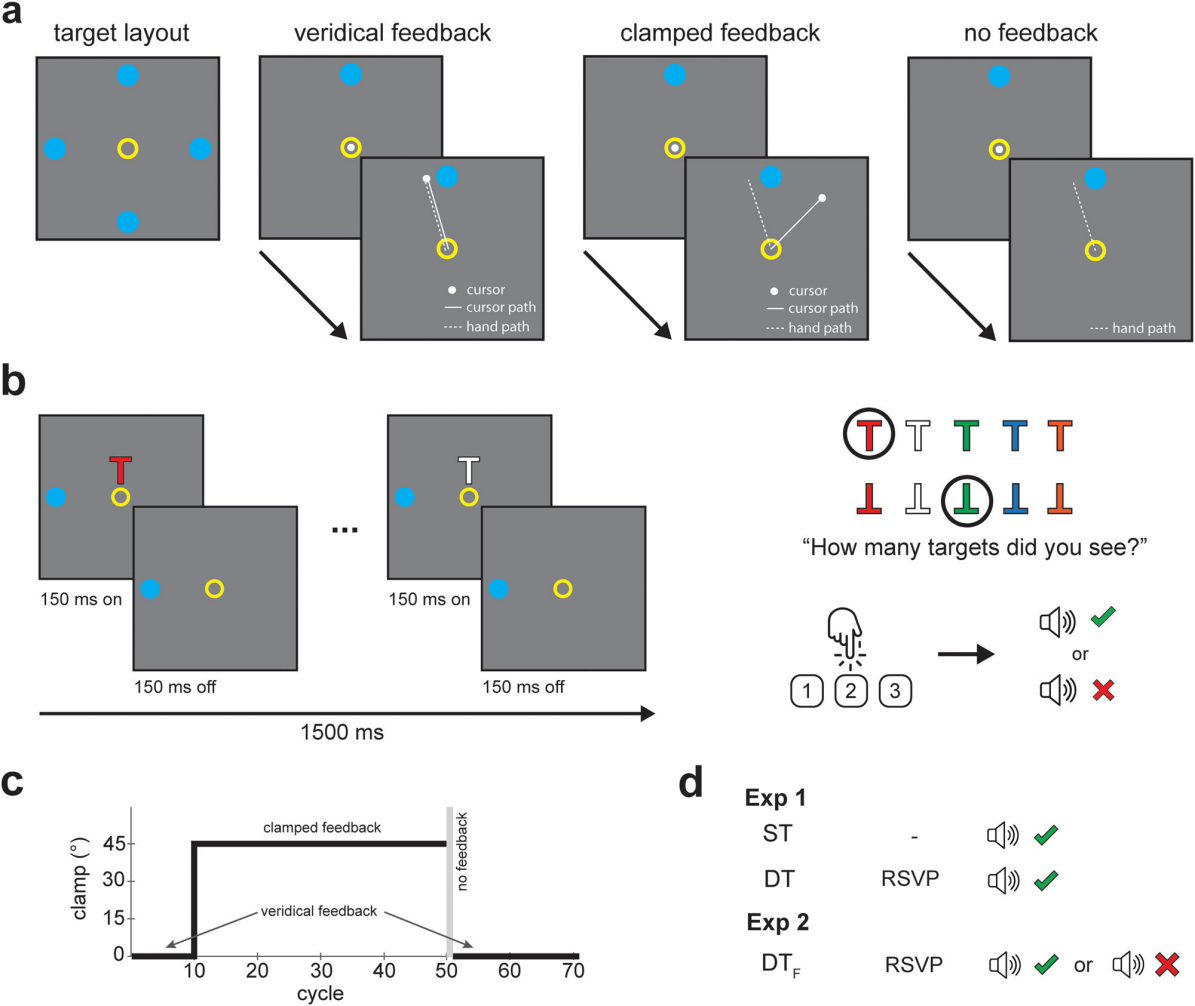
Experimental details. ***a***, In the reaching task, participants moved toward a target at one of four possible locations. Three forms of visual feedback were provided: veridical feedback, clamped feedback, and no feedback. ***b***, For the visual task, participants monitored a sequence of letter Ts consisting of color and orientation features that varied from trial-to-trial. Participants reported how many targets—upright red Ts and inverted green Ts—were presented each trial via keypress and received different forms of auditory feedback. ***c***, The reaching task was the same in Exp 1 and 2. The first 10 cycles (40 trials) used veridical feedback. The next 40 cycles (160 trials) used ±45° clamped feedback (counterbalanced). No feedback was provided for the first cycle (4 trials) after learning. ***d***, In Exp 1, there were two experimental groups: ST and DT. In Exp 2, there was one experimental group: dual task feedback (DT_F_).

Participants performed center-out reaching movements from a starting position (yellow annulus, 1° diameter) to a target (blue circle, 1° diameter) at a radial distance of 5 cm on a gray background (Fig. 1*a*). Targets appeared in the cardinal directions (0, 90, 180, 270°) in a pseudorandom order every four trials, which comprised a cycle. Hand position feedback was sometimes provided via a cursor (white circle, 0.25° diameter).

On each trial, the target appeared after a 1–2 s delay. Participants made “shooting” movements through the target. Instructions emphasized initiating movements immediately after the target appeared and minimizing corrective adjustments. See Appendix 2 (https://doi.org/10.5281/zenodo.18510924). Feedback was provided until the hand exceeded the target distance, where the cursor was frozen for 1 s. The target and cursor were then replaced with a white ring representing the distance from the starting position. The cursor reappeared when the hand was within 1 cm of the starting position, allowing the next trial to be initiated.

Three types of visual feedback were provided: veridical, clamped, and no feedback (Fig. 1*a*). In veridical feedback trials, the cursor was congruent with hand movement—for example, forward hand motion produced upward cursor motion. In clamped feedback trials, the cursor followed a fixed trajectory relative to the target, offset by 45° clockwise or counterclockwise (counterbalanced).

Participants were told that they could not control the cursor and were instructed to ignore it and move directly toward the target. Despite these instructions, participants reliably showed gradual changes in hand angle driven by SPE rather than task error or reward, which reflects implicit adaptation (Morehead et al., 2017; Kim et al., 2018). In no-feedback trials, the cursor was hidden; participants were instructed to move directly toward the target knowing that the cursor would not appear.

#### Visual task

In the DT conditions, participants performed a RSVP task while reaching. Each trial, a sequence of five Ts (0.75° diameter) appeared above the starting position (2.2° radius). Each T was shown for 150 ms, followed by a 150 ms blank, resulting in a 1,500 ms sequence that overlapped with the reaching task (Fig. 1*b*). The Ts varied in color (red, green, blue, orange, or white) and orientation (upright or inverted). Targets were defined by a conjunction of features: upright red Ts and inverted green Ts. Each trial contained 1, 2, or 3 targets. The number of targets, their identity, and location in the sequence were randomized each trial. For the distractors, color and orientation were completely randomized from the nontarget options.

Participants were told that the location and number of RSVP stimuli shown each trial were fixed but that the number of targets would vary. After each trial, they reported the number of targets via keypress. In Exp 1, participants received a high-pitched tone as noncontingent feedback for both correct and incorrect responses (DT), whereas in Exp 2, feedback was contingent on performance: correct responses elicited a high-pitched tone and incorrect responses a low-pitched tone (DT_F_; Fig. 1*b*).

In the ST condition, participants performed the same reaching task as the DT condition. To maintain a similar sequence of actions, they completed a prompted keypress (1, 2, or 3) after each trial. RSVP stimuli appeared on every trial to standardize visual input, but participants were told to ignore it.

### Procedures

#### Exp 1

Exp 1 was designed to examine the influence of a concurrent visual task on implicit adaptation. This was done by comparing implicit adaptation in individuals who only performed a reaching task (ST) to those who performed the same reaching task while also monitoring a changing stream of visual information at a fixed location (DT). The key difference between the experimental groups was that individuals in the DT group needed to attend to the RSVP stream while those in the ST group did not.

Participants performed the reaching task throughout the experiment. Practice was provided and consisted of 20 trials of reaching with veridical feedback, 20 trials of reaching with clamped feedback, and 20 trials of reaching with veridical feedback while performing the RSVP task. Participants completed this practice schedule regardless of their group assignment. During practice, the clamped feedback trials alternated between clockwise and counterclockwise directions to prevent cumulative learning while familiarizing participants with the task.

The experiment consisted of four stages: 40 trials of veridical feedback, 160 trials of clamped feedback, 4 trials of no feedback, and 80 trials of veridical feedback (Fig. 1*c*). The experiment was completed in ∼45 min without prolonged breaks. However, there were short pauses (30 s) when the visual feedback was manipulated to allow the experimenter to inform the participant about upcoming changes. Participants were randomly assigned to either ST or DT (Fig. 1*d*).

#### Exp 2

Exp 2 was designed to sustain engagement with the RSVP task using response-contingent feedback to determine whether findings observed in Exp 1 reflected reliable sensitivity of implicit adaptation to goal-directed attentional demands. To this end, we modified the RSVP task so that the feedback was contingent on participants’ responses (correct responses received a high-pitched tone, incorrect responses a low-pitched tone). The procedures were identical to Exp 1, except that only one experimental group was collected (Fig. 1*d*).

### Data analysis

#### Response data

Trial-by-trial responses were obtained from the prompted keypress in ST and from the RSVP task in DT and DT_F_. Accuracy was summarized as the proportion of correct responses within predefined analysis windows: baseline (cycles 6–10), onset (cycles 11–12), early learning (cycles 13–17), and late learning (cycles 46–50). The onset window captured a transient disruption following the introduction of clamped feedback; early and late learning windows matched those used for implicit adaptation.

#### Reaching data

Reaching data were processed in MATLAB (2024b). Hand position signals were low-pass filtered using an eighth-order Butterworth filter with a 10 Hz cutoff. Each trajectory was rotated to a common axis, corresponding to the 90° target (i.e., the 12 o’clock position). Reach onset was defined as the time point when hand velocity exceeded 1 cm/s; reach offset was defined when hand position exceeded 5 cm (i.e., target distance). These events were used to define reaction time (RT) as the duration to reach onset and movement time (MT) as the duration between reach onset to reach offset, both measured in milliseconds. Total action duration (TAD) was computed as RT + MT to verify that the reaching and RSVP tasks overlapped temporally.

Hand angle, measured in degrees, was defined as the angular offset between two vectors: one from the reach onset position to the target center and one from the reach onset position to the reach offset position. Angles were adjusted such that the direction of clamped feedback was counterclockwise relative to the target. Positive hand angle values denote clockwise deviation, such that positive changes in the hand angle were expected during exposure to clamped feedback.

The hand angle was averaged over four successive trials (i.e., cycle), one per target location. Trials were excluded if any of the following criteria were met: (1) movement amplitude was less than 5 cm; (2) hand angle exceeded ±90° or deviated >25° from a 10-point moving median; (3) RT exceeded the 97.5th percentile of the RT distribution, obtained via kernel density estimation; or (4) MT exceeded 500 ms. The median number of excluded trials per participant was 8 (3.9%), ranging from 1 (0.5%) to 22 (10.8%). In total, 640 trials (4.4%) were excluded. No cycle had all four trials removed.

At baseline, the mean hand angle across the last five cycles (6–10) ranged from −1.4 to 3.7°. Group means and standard deviations were quantified using Bayesian estimation with a Student's *t* likelihood. Groups showed no credible differences (ST, median = 1.04°; 89% HDI [0.54, 1.56]; DT, 0.49°, [0.14, 0.83]; DT_F_, 0.55°, [0.17, 0.94]). Learning and aftereffect cycles (cycles 11–51) were baseline-adjusted by subtracting each participant's mean hand angle during the last five cycles (6–10). This ensured that measures of implicit adaptation reflected changes from each participant's starting point.

#### Modeling

We modeled implicit adaptation using a state-space model rather than a simple exponential function because the former provides a mechanistic account of how behavior evolves over time. This formulation allows separate estimation of retention and error sensitivity parameters and affords prediction under unobserved conditions, whereas exponential fits are purely descriptive.

Hand angle data were modeled using a single-rate state–space model (Smith et al., 2006; Kim et al., 2019), in which the internal state *x_n_* represents the learner's estimate of the visuomotor mapping between hand movement and visual feedback on trial *n*. The internal state evolves according to the following:

$$x_{n+1}=Ax_{n}+be_{n},$$

where *A* is the retention parameter, *b* is the error sensitivity parameter, and *e_n_* is the SPE on trial *n.* On rotation trials, $e_{n}=r_{n}-y_{n}$, that is, the difference between the imposed visuomotor rotation *r_n_* and the motor output *y_n_*. Veridical feedback trials (where *r_n_* = 0) thus represent a special case of a rotation trial. On error-clamp trials, $e_{n}=c_{n}$, a fixed angular offset that does not depend on the motor output *y_n_*.

The motor output is given by as follows:

$$y_{n}=-x_{n}+\varepsilon ,\;\;\varepsilon ∼N(0,\sigma^{2}),$$

with ε representing measurement and motor output noise. To account for time-dependent forgetting during extended pauses, we included a set break decay parameter *d*. If a set break occurred between trial *n* and trial *n* + 1, the retention factor became *A^d^* (instead of *A*) for the next trial, effectively increasing forgetting (Albert and Shadmehr, 2018). This prevents the learning parameters from having to account for this variance.

Model parameters were estimated using constrained nonlinear optimization (*fmincon*) to minimize the mean-squared error (MSE) between predicted and observed hand angle. Parameter bounds were set to A∈[0.01,0.999], b∈[0.001,0.75], and d∈[1,2], ensuring estimates fell within plausible ranges. Each participant’s data were fit from 200 random initializations uniformly sampled from within these bounds. Final parameter estimates were taken from the run yielding the lowest MSE. The initial state was fixed at *x_n_* = 0 because the data were baseline-adjusted, and the noise variance $\sigma^{2}$ was not explicitly estimated, as it was captured by the residual MSE.

The reported results are based on cycle-level data. Trial-level estimates were nearly identical, following the expected relationship: $A_{\mathrm{cycle}}=A_{\mathrm{trial}}^{4}$ and $b_{\mathrm{cycle}}=4b_{\mathrm{trial}}$. Agreement between cycle- and trial-level estimates was quantified using Bayesian estimation to obtain the concordance correlation coefficient (Lin, 1989), which indicated excellent agreement for both retention (*ρ_c_* = 0.96, 89% HDI [0.95, 0.97]) and error sensitivity (*ρ_c_* = 0.98, 89% HDI [0.97, 0.99]).

### Statistical analysis

Statistical analyses were conducted in R (v4.5.0) using Bayesian estimation with Stan, either via rstan (v2.32.7) or brms (v2.22.0). Point estimates reflect the posterior median, and uncertainty is summarized using the 89% highest density interval (HDI). The 89% HDI offers a narrower and more interpretable summary than the conventional 95% interval, while still capturing the most credible range of parameter values, which is advantageous in small-sample experiments and exploratory research (McElreath, 2018). All uncertainty intervals reflect the 89% HDI unless otherwise indicated.

Priors were specified to be weakly or moderately informative, depending on the variable and model structure. For example, priors on group means were centered on observed sample statistics with wide dispersion (e.g., ±30° for the hand angle), and priors on variance parameters followed inverse gamma distributions scaled to observed variability. Complete model specifications, including prior distributions and likelihood functions, are documented in Appendix 3 (https://doi.org/10.5281/zenodo.18510924).

Models were fitted using the No-U-Turn Sampler with 4 chains of 3,500 iterations each (1,000 warm-up per chain). Identical sampling settings (adapt_delta ≥ 0.9; max_treedepth ≥ 10) were applied across models. For all models, we verified convergence using standard diagnostics, including R̂ values, high effective sample sizes, no divergent transitions, and acceptable Bayesian Fraction of Missing Information, consistent with recent recommendations (Baribault and Collins, 2025).

We sometimes report posterior probabilities for directional claims [e.g., Pr(DT > ST) = 0.95], which represent the probability that the effect favors a particular direction given the data and model. Unlike *p* values, these probabilities are direct statements about model parameters or functionals. We also report Type S (sign) and Type M (magnitude) errors to characterize directional reliability and potential exaggeration of estimated effects (Gelman and Carlin, 2014). However, our implementation marginalizes over posterior uncertainty rather than conditioning on a point estimate. See Appendix 3 for details (https://doi.org/10.5281/zenodo.18510924).

### Code Accessibility

Data, computational modeling code, statistical analysis code, and figure-generation code are freely available online at https://doi.org/10.5281/zenodo.18510924. Analyses were conducted in MATLAB (R2024b) and R (v4.5.0) using Bayesian estimation with rstan (v2.32.7) and brms (v2.22.0). All analyses were performed on a Windows 11 PC. The repository includes extended methodological documentation: data exclusion (Appendix 1), experimental instructions (Appendix 2), and detailed statistical analyses (Appendix 3).

## Results

### Exp 1: implicit adaptation is transiently modulated by goal-directed attention

This experiment tested whether attentional demands imposed by a concurrent visual task modulate the expression of implicit adaptation. Participants in ST reached toward the target while ignoring the RSVP stream and then responded to a simple prompt (1, 2, or 3). Participants in DT monitored the RSVP stream while reaching and reported the number of targets seen (also 1, 2, or 3).

#### Attentional demands transiently enhanced implicit adaptation

**Figure 2.**
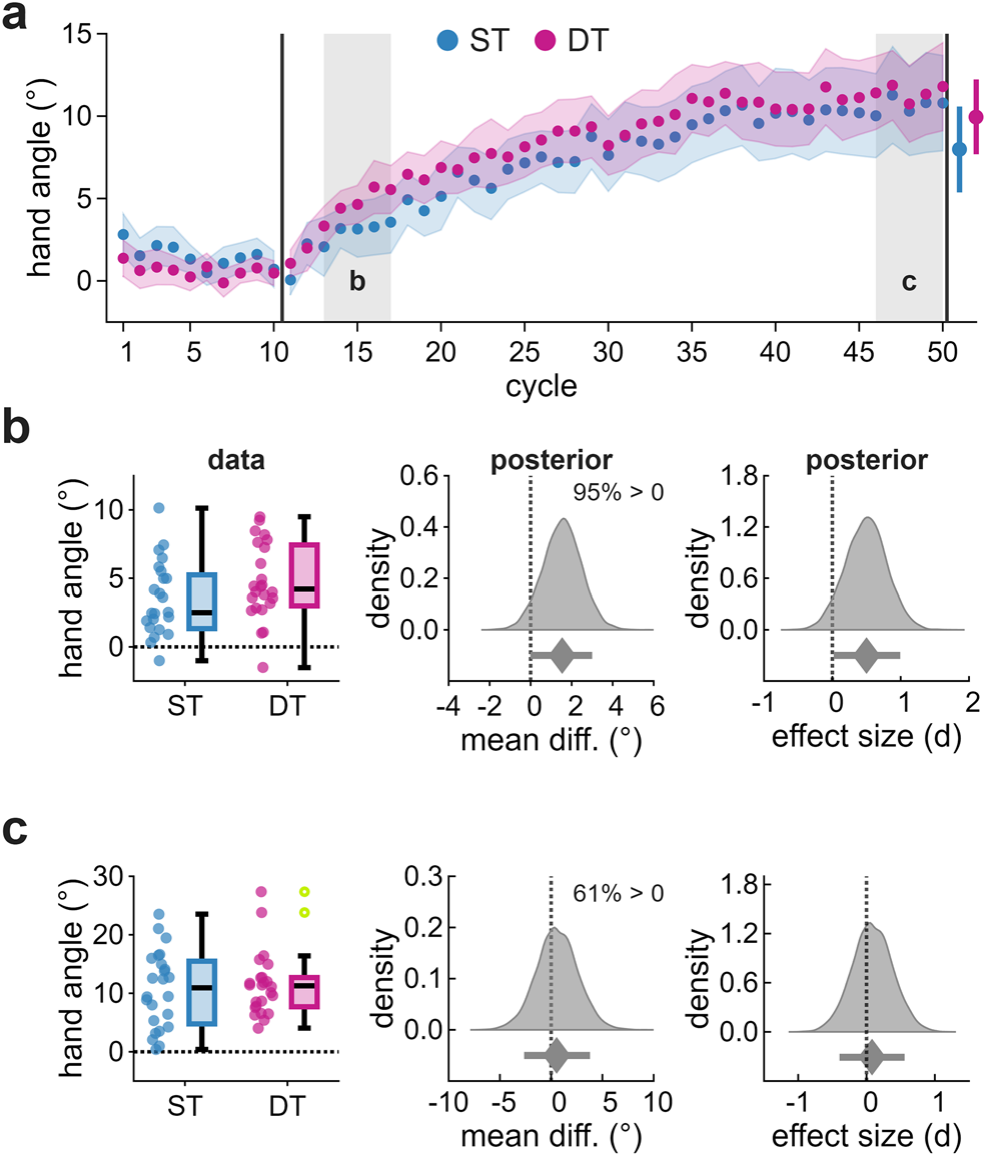
Implicit adaptation in Exp 1. ***a***, Hand angle across cycles for ST (blue) and DT (magenta). Dots indicate group means; shaded regions indicate ±2 SEM. Gray-shaded regions denote predefined analysis windows: (***b***) early learning and (***c***) late learning. Left panels show individual data points and box plots. Open circles indicate individual data points beyond the boxplot whiskers (>1.5× IQR), which remain included in all analyses. Middle and right panels display posterior distributions for the group mean difference (middle) and standardized effect size (right), with posterior medians (diamonds) and 89% HDIs (error bars). Text annotations indicate the proportion of posterior mass above zero.

To induce implicit adaptation, participants were exposed to clamped feedback while monitoring the RSVP stream (DT) or ignoring it (ST). Exposure to clamped feedback produced an incremental shift in hand angle in the direction opposite the clamp, consistent with implicit adaptation (Fig. 2*a*). Group comparisons were conducted within predefined windows: early learning (cycles 13–17), late learning (cycles 46–50), and the aftereffect (cycle 51).

During early learning (Fig. 2*b*), implicit adaptation was modestly enhanced in DT. The mean hand angle was 3.2° [2.0, 4.3] for ST and 4.7° [3.7, 5.7] for DT, yielding a mean difference of 1.5° [0.0, 3.0] and a standardized effect size of 0.50 [0.01, 0.98]. The posterior probability that DT exceeded ST was 95%, providing clear directional evidence for enhanced adaptation, though the magnitude of this enhancement remained uncertain. Type S and M error estimates (11% and 1.07, respectively) suggest this directional effect would likely replicate.

By late learning (Fig. 2*c*), the hand angle showed an appreciable positive shift, with considerable individual differences (range, 0.4–27.3°). At the group level, implicit adaptation was 10.7° [8.2, 13.1] for ST and 11.2° [9.2, 13.2] for DT. The mean difference of 0.5° [−2.6, 3.8] and standardized effect size of 0.08 [−0.39, 0.56] indicated negligible group differences. Directional uncertainty was greater than during early learning, with only 61% probability that DT exceeded ST, consistent with convergence of the learning trajectories.

Following the removal of clamped feedback, participants completed four no-feedback trials (Cycle 51). The mean aftereffect was 8.1° [5.8, 10.5] for ST and 9.7° [7.7, 11.7] for DT, with a mean difference of 1.6° [−1.5, 4.7] and an effect size of 0.25 [−0.22, 0.74]. Although the magnitude of this difference mirrored that observed during early learning, directional uncertainty was greater (79% probability that DT exceeded ST), providing weak evidence of a group difference.

In summary, implicit adaptation was consistently expressed across participants and showed modest but transient enhancement while performing a concurrent visual task. This occurred despite identical visual displays across conditions. Group differences emerged early but diminished, with learning trajectories converging by the end of learning.

#### RSVP performance and its relationship to implicit adaptation

**Figure 3.**
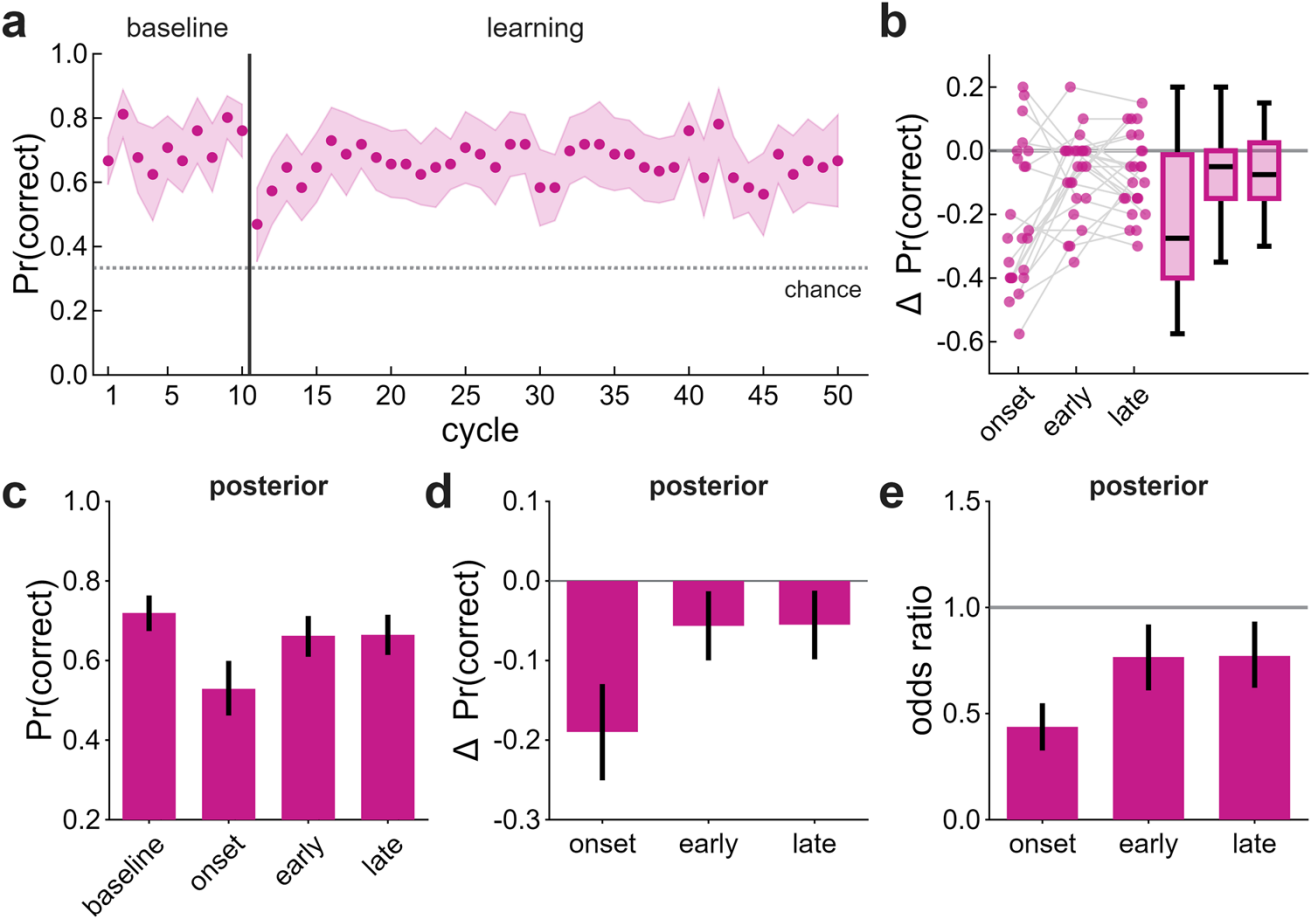
DT performance in Exp 1. ***a***, Proportion of correct responses across baseline and learning. Dots indicate group means; shaded regions indicate ±2 SEM. The dotted line is chance performance (0.33). ***b***, Individual participant changes in accuracy relative to baseline (cycles 6–10) for each learning epoch: onset (cycles 11–12), early (cycles 13–17), and late (cycles 46–50). ***c–e***, Posterior summaries from the hierarchical binomial model showing (***c***) median accuracy, (***d***) changes from baseline, and (***e***) odds ratios relative to baseline. Error bars are 89% HDIs. See also Extended Data Figure 3-1 for ST.

We examined RSVP performance to confirm task engagement and to test whether implicit adaptation and the visual task competed for cognitive resources. In DT, accuracy was above-chance, confirming engagement with the RSVP task (Fig. 3*a*). However, accuracy dropped sharply following clamp onset (Fig. 3*b*). In ST, accuracy remained high throughout (Extended Data Fig. 3-1).

10.1523/ENEURO.0243-25.2026.f3-1 Figure 3-1 **Single-task (ST) performance in Exp 1**. (a) Proportion of correct responses across baseline and learning. Dots indicate group means; shaded regions indicate ± 2 SEM. The dotted line is chance performance (0.33). Accuracy remained well above chance throughout the experiment. (b) Individual changes in accuracy relative to baseline for each learning epoch: onset (cycles 11–12), early (cycles 13–17), and late (cycles 46–50). (c–e) Posterior summaries from a hierarchical binomial model showing (c) median accuracy, (d) changes from baseline, and (e) odds ratios relative to baseline. Error bars are 89% HDIs. Changes were centered near zero, indicating that performance was not systematically affected by the clamped feedback. Download Figure 3-1, TIF file.

RSVP accuracy was modeled using hierarchical binomial regression, which indicated a decline from 0.72 [0.67, 0.76] to 0.53 [0.46, 0.60] before partially recovering during early (0.66 [0.61, 0.71]) and late (0.66 [0.61, 0.71]) learning (Fig. 3*c–e*). This disruption was time-locked to the onset of clamped feedback, despite instructions to ignore the cursor.

The co-occurrence of RSVP disruption and enhanced implicit adaptation raises the possibility of a resource trade-off: participants whose RSVP accuracy declined more may show greater implicit adaptation. However, changes in RSVP accuracy were unrelated to implicit adaptation magnitude at all epochs (onset–early, *r* = −0.16 [−0.47, 0.14]; early–early, *r* = −0.16 [−0.47, 0.15]; late–late, *r* = −0.01 [−0.33, 0.30]). Individual differences in RSVP accuracy were unrelated to the magnitude of implicit adaptation, providing no evidence of a resource trade-off.

### Exp 2: RSVP feedback sustains the enhancement of implicit adaptation

In Exp 1, RSVP accuracy dropped sharply at learning onset and remained below baseline. The enhancement of implicit adaptation was transient, diminishing as learning progressed. Previous work shows that motivational factors, including feedback and reward, can enhance engagement and sustain attention (Sarter et al., 2006; Botvinick and Braver, 2015; Yee and Braver, 2018). In Exp 2, we examined whether providing trial-by-trial RSVP feedback (DT_F_) would maintain RSVP performance and potentially sustain the enhancement of implicit adaptation.

#### Feedback sustains RSVP engagement, but it remains independent of implicit adaptation

**Figure 4.**
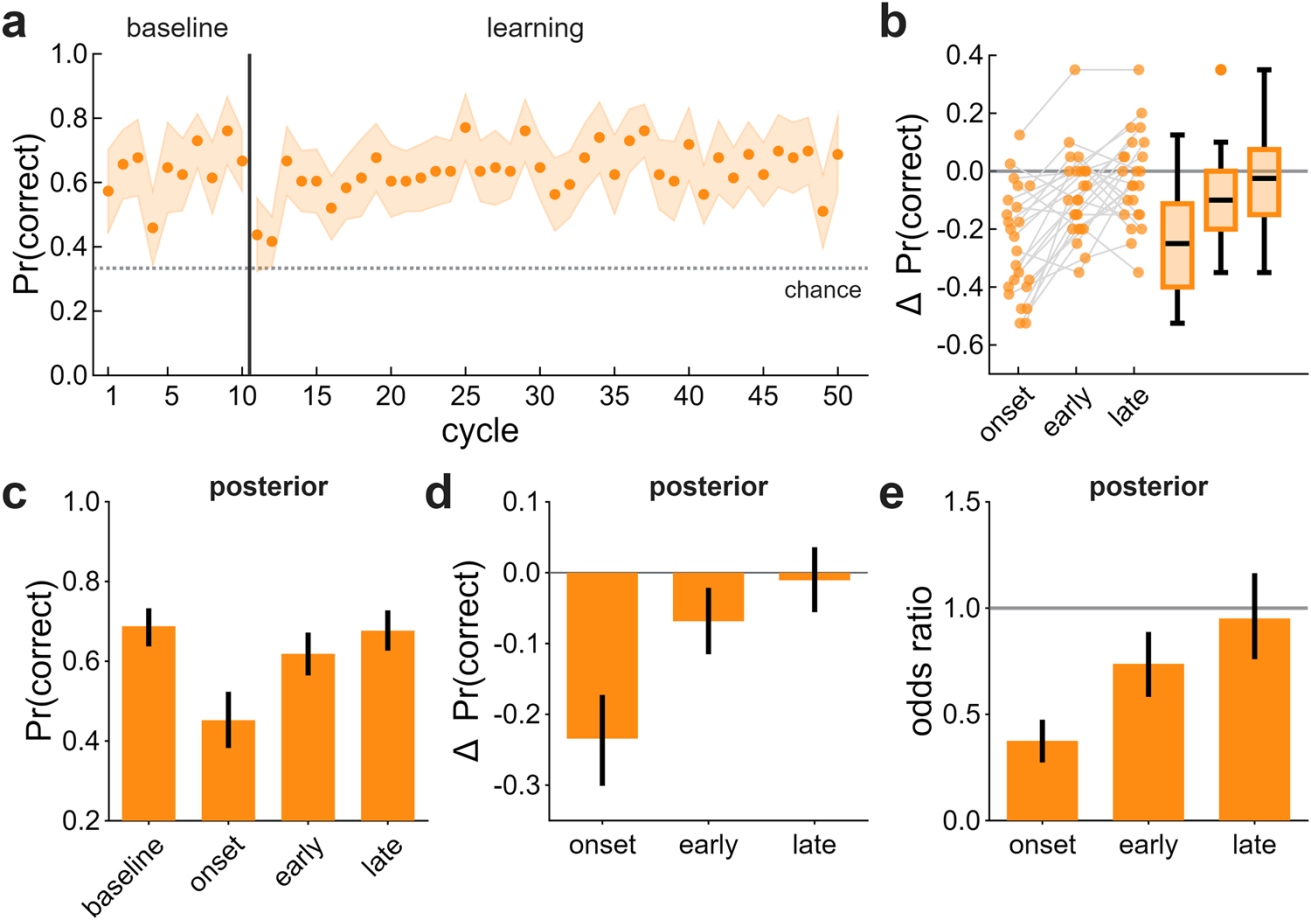
Dual-task (DT_F_) performance in Exp 2. ***a***, Proportion of correct responses across baseline and learning. Dots indicate group means; shaded regions denote ±2 SEM. The dotted line is chance performance (0.33). ***b***, Individual changes in accuracy relative to baseline (cycles 6–10) for each learning epoch: onset (cycles 11–12), early (cycles 13–17), and late (cycles 46–50). ***c–e***, Posterior summaries from the hierarchical binomial model showing (***c***) median accuracy, (***d***) changes from baseline, and (***e***) odds ratios relative to baseline. Error bars are 89% HDIs. See also Extended Data Figure 4-1 for comparison to DT.

We examined whether response-contingent feedback would sustain RSVP performance and prevent the incomplete recovery observed in Exp 1 (Fig. 4). As in Exp 1, accuracy dropped sharply at clamp onset (Δ = −0.23 [−0.30, −0.17]). However, unlike Exp 1, accuracy fully recovered by late learning (Δ = −0.01 [−0.06, 0.04]). Direct comparisons between DT and DT_F_ at each epoch were not credible (Extended Data Fig. 4-1), but the recovery patterns differed: DT_F_ returned to baseline, whereas DT did not. This suggests response-contingent feedback sustained RSVP engagement. The persistence of the onset disruption in both groups indicates that it likely reflects attentional capture. By this we mean, the error clamp acts as a salient distractor despite knowledge that it is task-irrelevant, which draws attention away from the RSVP stream.

10.1523/ENEURO.0243-25.2026.f4-1 Figure 4-1 **Comparison of dual-task performance (DT and DT_F_)**. (a) Proportion of correct responses across baseline and learning. Dots indicate group means; shaded regions represent ± 2 SEM. The dotted line indicates chance performance (0.33). (b) Posterior median accuracy with 89% HDIs for each group and epoch. (c) Posterior median group differences (DT_F_ – DT) with 89% HDIs. Group differences were small and spanned zero at all epochs. Download Figure 4-1, TIF file.

As in Exp 1, RSVP accuracy was unrelated to implicit adaptation at any epoch (onset, *r* = −0.08 [−0.39, 0.24]; early, *r* = −0.06 [−0.37, 0.26]; late, *r* = 0.16 [−0.15, 0.48]). Collapsing across DT groups did not alter this pattern (onset, *r* = −0.11 [−0.33, 0.12]; early, *r* = −0.11 [−0.33, 0.12]; late, *r* = 0.08 [−0.14, 0.30]). Thus, the independence of RSVP performance and implicit adaptation observed in Exp 1 replicated in Exp 2.

#### Implicit adaptation remained elevated throughout learning

**Figure 5.**
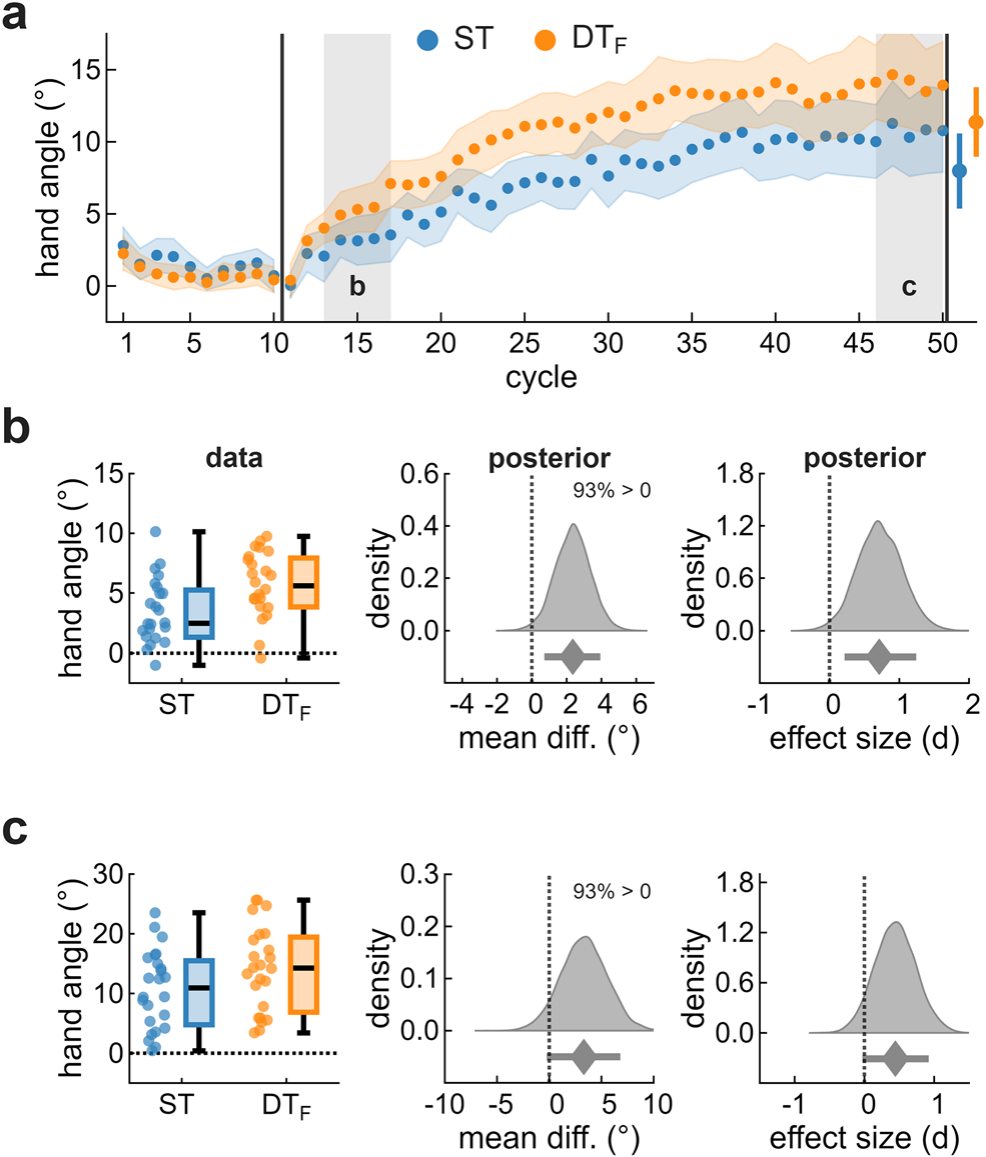
Implicit adaptation in Exp 2. ***a***, Hand angle across cycles for ST (blue) and DT_F_ (orange). Dots represent group means; shaded regions indicate ±2 SEM. Gray-shaded regions denote predefined analysis windows for (***b***) early learning and (***c***) late learning. Left panels show individual data with box plots. Middle and right panels show posterior densities for the group mean difference and standardized effect size, with posterior medians (diamonds) and 89% HDIs (error bars). Text annotations indicate the posterior probability that DT_F_ exceeded ST. See also Extended Data Figure 5-1 (movement kinematics), Extended Data Figure 5-2 (bootstrap analysis), and Extended Data Figure 5-3 (sensitivity analysis).

To examine whether increased RSVP engagement influenced implicit adaptation, we compared the DT_F_ group (Exp 2) with the ST and DT groups (Exp 1). DT_F_ and ST diverged early and remained separated throughout learning (Fig. 5*a*). Group comparisons were again conducted within predefined windows: early learning (cycles 13–17), late learning (cycles 46–50), and the aftereffect (cycle 51).

10.1523/ENEURO.0243-25.2026.f5-1 Figure 5-1 **Reaction time (RT), movement time (MT), and total action duration (TAD).** Each dot represents an individual participant during learning (cycles 11-50). Left panels: data with group means ±2 SEM. Right panels: posterior medians and 89% HDIs. (a) RT was short (440 ms [428, 454]) and stable across learning, with no systematic group differences. (b) MT was also short (184 ms [175, 192]), with no evidence of change over learning, nor any group differences. (c) TAD was consistently less than 1500 ms (629 ms [612, 648]), indicating that the reaching and visual task were executed in parallel. Download Figure 5-1, TIF file.

10.1523/ENEURO.0243-25.2026.f5-2 Figure 5-2 **Bootstrap estimation of group differences in implicit adaptation**. (a) Mean hand angle across cycles for ST, DT, and DT_F_. Shaded regions denote 89% bootstrap HDIs. (b) Pairwise differences (Δ hand angle) between groups across cycles, with shaded regions indicating 89% HDIs. To reduce cycle-to-cycle noise, participant-level data were smoothed with a five-cycle centered moving average before resampling. Group means and pairwise differences were estimated via bootstrap resampling (10,000 iterations), and 89% HDIs were computed for each cycle. We interpret differences as credible when the 89% HDI for Δ hand angle excluded 0° (asterisks), particularly across contiguous cycles, reflecting a sustained rather than transient divergence. This provides a continuous, uncertainty-based estimate of when and by how much groups differed, serving as an estimation-based alternative to cluster-based permutation tests. The latter are designed to control long-run family-wise error rates across hypothetical replications and are therefore conservative for estimation-focused analyses where an exact replication is rarely conducted. Download Figure 5-2, TIF file.

10.1523/ENEURO.0243-25.2026.f5-3 Figure 5-3 **Sensitivity analysis for predefined analysis windows.** Adjacent five-cycle windows were examined to assess whether group differences depended on window selection. For early learning, windows were shifted forward; for late learning, windows were shifted backward. Asterisks (*) indicate the predefined windows reported in the main text. Results were stable across windows, confirming that the predefined windows provided conservative estimates. Download Figure 5-3, DOCX file.

During early learning (Fig. 5*b*), the mean hand angle for DT_F_ was 5.5° [4.4, 6.6], exceeding both Exp 1 groups (ST, 3.2° [2.0, 4.3]; DT, 4.7° [3.7, 5.7]). The difference between DT_F_ and ST was 2.4° [0.7, 3.9], with an effect size of 0.72 [0.22, 1.25]. The posterior probability that DT_F_ exceeded ST was 99%, with a sign error rate of 5% and exaggeration ratio of 1.02. The DT_F_–DT difference was smaller and less certain (0.8° [−0.7, 2.3]; *d* = 0.27 [−0.23, 0.76]), with 81% probability that DT_F_ exceeded DT. These results replicate the early enhancement observed in Exp 1, extending it to DT_F_.

During late learning (Fig. 5*c*), implicit adaptation remained elevated in DT_F_ at 14.0° [11.5, 16.6], compared with ST (10.7° [8.2, 13.1]) and DT (11.2° [9.2, 13.2]). The DT_F_–ST difference was 3.3° [−0.2, 6.8], with an effect size of 0.44 [−0.02, 0.93]. The posterior probability that DTF exceeded ST was 93%, with a sign error rate of 12% and exaggeration ratio of 1.09. The DT_F_–DT difference was smaller (2.8° [−0.4, 6.3]; *d* = 0.41 [−0.06, 0.89]), with 91% probability that DT_F_ exceeded DT. Although credible intervals included zero, the posterior distributions were predominantly positive, suggesting the early enhancement persisted. Increased between-subject variability likely reduced precision.

The aftereffect provided further support for increased implicit adaptation in DT_F_. The aftereffect for DT_F_ was 11.3° [9.2, 13.4], compared with ST (8.1° [5.8, 10.5]) and DT (9.7° [7.7, 11.7]). The DT_F_–ST difference was 3.2° [0.1, 6.4], with an effect size of 0.47 [−0.01, 0.94]. The posterior probability that DT_F_ exceeded ST was 95%, with a sign error rate of 11% and exaggeration ratio of 1.07. The DT_F_–DT difference was smaller and less certain (1.6° [−1.3, 4.6]; *d* = 0.26 [−0.20, 0.75]), with 81% probability that DT_F_ exceeded DT.

Together, these results indicate that implicit adaptation was consistently greater in DT_F_ than ST, with group differences emerging early and persisting throughout learning. Providing RSVP feedback both sustained RSVP engagement and enhanced implicit adaptation rather than attenuating it or leaving it unchanged.

#### Confirmatory checks

We verified that reaching kinematics were consistent with implicit adaptation and that the reaching and visual task overlapped temporally (Extended Data Fig. 5-1). RT was rapid (440 ms [428, 454]) and MT was short (184 ms [175, 192]), with no credible group differences or changes across learning. Reaches were consistently straight (straightness ratio = 0.996, [0.977, 0.999]). The tasks were performed in parallel: TAD was well below the 1,500 ms trial duration (629 ms [612, 648]). Leave-one-out cross-validation indicated that group-specific parameters did not improve model fits. Together, these results suggest that the observed group differences in implicit adaptation are not due to explicit reaiming, online correction, or sequential task execution.

To verify that the findings did not depend on the window selection, we conducted a continuous bootstrap analysis (Extended Data Fig. 5-2), which confirmed the time course of group differences, and sensitivity analyses across adjacent windows (Extended Data Fig. 5-3). For early learning, shifting windows forward yielded progressively larger effects, confirming our analyses were conservative; for late learning, adjacent windows indicated similar convergence or divergence patterns depending on the chosen contrast.

### Modeling: learning parameters reflect attentional demands

While behavioral measures revealed group differences in implicit adaptation, they cannot reveal how the underlying learning process was affected. Behavior reflects the aggregate output of two processes: trial-to-trial retention and error-driven updating. Similar learning trajectories can arise from different combinations of these mechanisms.

To decompose these contributions, we fit a single-rate state–space model that separately estimates retention (A) and error sensitivity (b). Retention governs how much of the internal state carries forward from one trial to the next. Error sensitivity determines how strongly the system updates in response to SPEs.

We modeled the group-level distributions of these parameters jointly, estimating both the means and the correlation between A and b within each condition. This allowed us to ask whether the observed group differences reflect changes in retention, error sensitivity, or their relationship.

#### Error sensitivity increased under attentional demands

**Figure 6.**
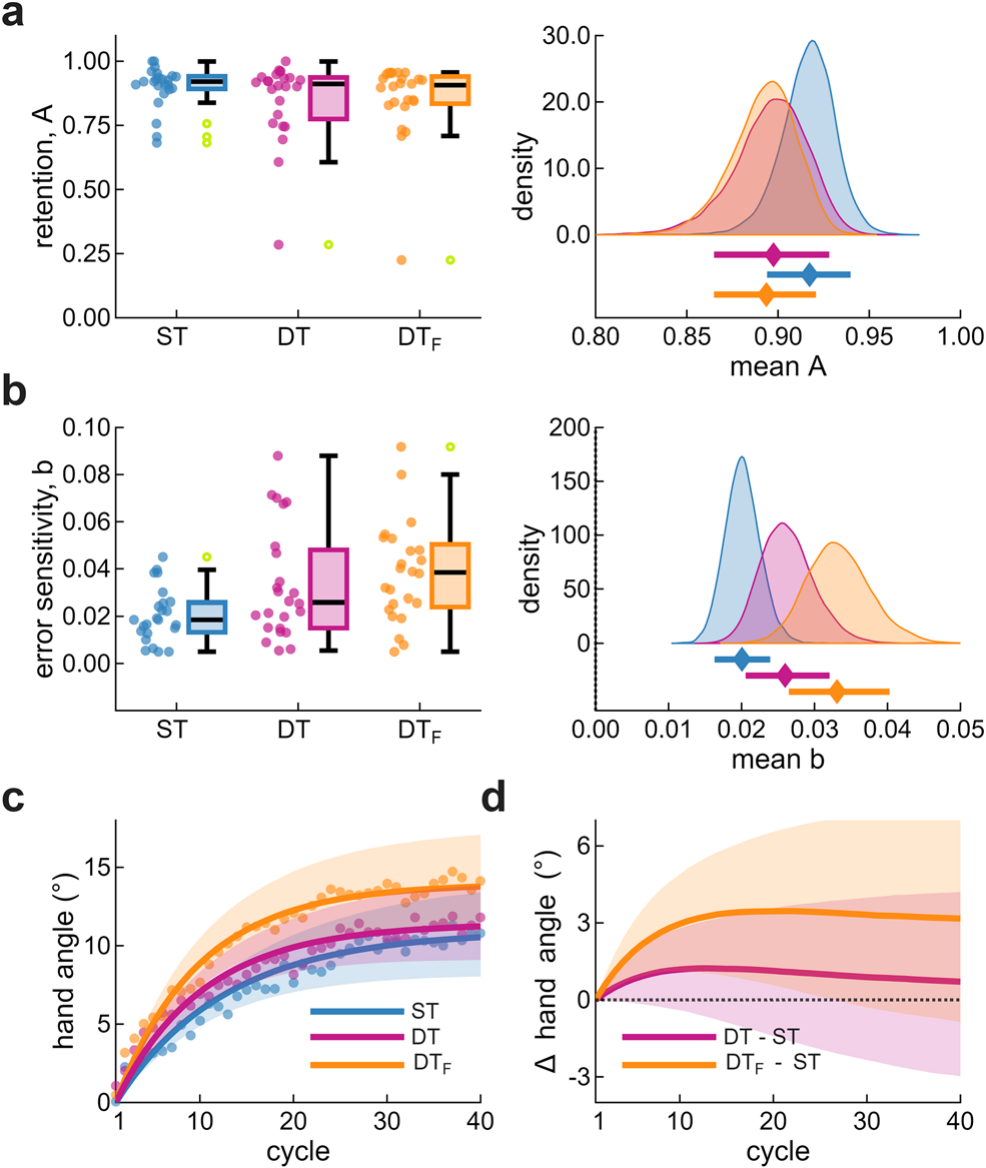
State-space model of implicit adaptation. ***a***, Retention and (***b***) error sensitivity. Retention is the proportion of the internal state preserved across cycles; error sensitivity reflects the magnitude of updating in response to SPEs. Left panels, Individual participant data and box plots. Open circles indicate individual data points beyond the boxplot whiskers (>1.5× IQR), which remain included in all analyses. Right panels, Posterior distributions of group means with medians (diamonds) and 89% HDIs (error bars). ***c***, Posterior predictive simulations from joint samples of A and b. Dots show empirical means; lines show posterior medians; shaded regions show 89% HDIs. ***d***, Predicted differences from ST across cycles, with shaded regions showing 89% HDIs. See also Extended Data Figure 6-1 for an engagement-modulated error sensitivity model.

Retention was high and similar across groups (Fig. 6*a*). Posterior medians were 0.917 [0.893, 0.940] for ST, 0.897 [0.864, 0.927] for DT, and 0.893 [0.864, 0.920] for DT_F_. Compared with ST, both DT conditions showed small reductions (DT, −0.020 [−0.061, 0.019]; DT_F_, −0.024 [−0.062, 0.012]), though these differences were uncertain and the DT–DT_F_ difference was negligible (−0.004 [−0.046, 0.040]).

10.1523/ENEURO.0243-25.2026.f6-1 Figure 6-1 **Learning trajectories under engagement-modulated error sensitivity**. (a) Simulated hand angle across cycles for ST, DT, and DT_F_. All simulations used identical retention (A = 0.92) and baseline error sensitivity (b = 0.02); conditions differed only in an engagement multiplier, m(n), that scaled error sensitivity on each cycle. For ST, m = 1 throughout. Two DT scenarios are shown: an initial boost that decays back to baseline (solid magenta; m(n) = 1 + 0.35·0.9ⁿ) and a partial but sustained boost (dotted magenta; m = 1.10). For DT_F_, engagement was elevated and sustained throughout (orange; m = 1.35). (b) Predicted differences from ST. The decaying engagement scenario for DT produces transient enhancement followed by convergence, matching the observed pattern. Any sustained elevation would predict persistent separation from ST, inconsistent with Exp 1. Sustained engagement in DT_F_ produces persistent enhancement, as observed in Exp 2. Download Figure 6-1, TIF file.

Error sensitivity showed a graded increase from ST to DT to DT_F_ (Fig. 6*b*). Posterior medians were 0.020 [0.016, 0.024] for ST, 0.026 [0.021, 0.032] for DT, and 0.033 [0.026, 0.040] for DTF. Compared with ST, DT showed a modest increase (0.006 [−0.001, 0.013]), while DT_F_ showed a larger, more reliable increase (0.013 [0.005, 0.021]). The DT_F_–DT difference was smaller and less certain (0.007 [−0.002, 0.016]). The probability that error sensitivity was higher in DT_F_ than ST was >0.99; for DT exceeding ST, the probability was 0.93; for DT_F_ exceeding DT, the probability was 0.90. The posterior probability of the monotonic ordering (ST < DT < DT_F_) was 0.83—nearly 5 : 1 odds in favor of a graded change in error sensitivity.

To illustrate how these parameter differences shape behavior, we simulated posterior predictive trajectories from the joint distributions of A and b (Fig. 6*c*). The simulated learning curves closely matched the empirical data, confirming that modest shifts in error sensitivity and retention reproduce the observed trajectories. Predicted differences from ST show early enhancement for both DT groups, with DT converging toward ST while DT_F_ maintains separation throughout learning (Fig. 6*d*). The widening uncertainty reflects the substantial individual differences in steady-state adaptation, highlighting the need for within-subject designs to more precisely characterize individual-level effects. Finally, RSVP accuracy was not associated with retention or error sensitivity, reinforcing the independence of the RSVP performance and implicit adaptation.

#### Retention and error sensitivity traded off under attentional demands

**Figure 7.**
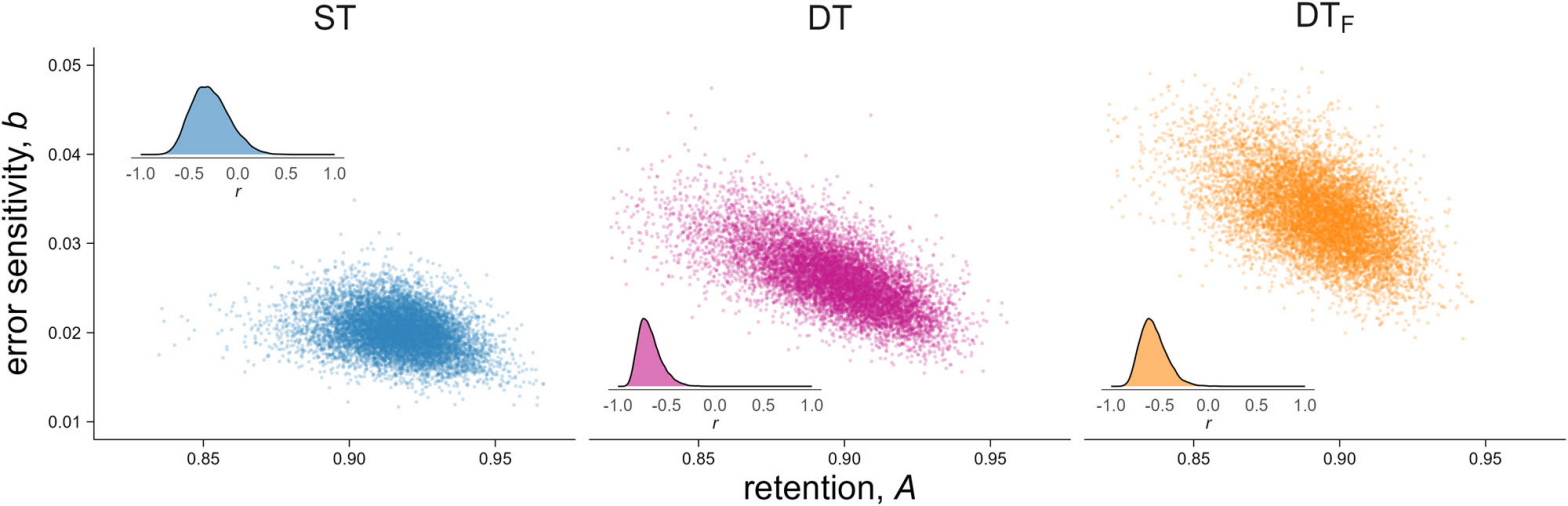
Joint posterior distributions of retention (***a***) and error sensitivity (***b***). Each panel shows posterior samples from the group-level means of A and b, illustrating both the location and covariance structure. Insets display posterior distributions of the correlation between A and b. See also Extended Data Figure 7-1 for parameter recovery simulations.

We next asked whether retention and error sensitivity covary differently across conditions (Fig. 7). In ST, the correlation was negative but weak and uncertain (*r* = −0.30 [−0.60, 0.02]). In contrast, both DT conditions showed strong negative correlations: DT (*r* = −0.68 [−0.85, −0.50]) and DT_F_ (*r* = −0.58 [−0.78, −0.37]). Thus, under attentional demands, individuals who retained more from trial to trial tended to respond less to errors.

10.1523/ENEURO.0243-25.2026.f7-1 Figure 7-1 **Parameter recovery simulation table**. A potential concern is that negative correlations between retention (A) and error sensitivity (b) can arise as an artifact of model fitting, since different parameter combinations can produce similar steady-state learning. To rule this out, we sampled A and b independently from the group-level posterior distributions (breaking any correlation; expected *r* = 0), simulated 1,000 learning trajectories per group with measurement noise drawn from the observed residual distributions, and refit the model. We then computed Pearson correlations on the recovered parameter estimates. Simulated *r* reflects independent sampling (expected *r* = 0). Recovered *r* reflects bias introduced by model fitting (median [89% HDI] across simulated experiments). Observed *r* is the Pearson correlation from participant-level point estimates. Pr(observed ≤ recovered) indicates the proportion of recovered correlations at least as extreme as observed. The fitting procedure introduced modest negative bias. For ST, the observed correlation was compatible with fitting artifact. For DT and DT_F_, the observed correlations exceeded nearly all recovered correlations, indicating genuine individual differences rather than model degeneracy. Download Figure 7-1, DOCX file.

A potential concern is that negative A–b correlations are an artifact of model fitting. To rule this out, we conducted parameter recovery simulations, sampling A and b independently from the posterior and refitting the model (Extended Data Fig. 7-1). The fitting procedure introduced modest negative bias, but the observed correlations in DT and DT_F_ exceeded nearly all recovered correlations (>99%). For ST, the observed correlation was compatible with fitting artifact. Thus, the trade-off in learning parameters under DT conditions reflects genuine individual differences, not model degeneracy.

## Discussion

Implicit adaptation is an automatic learning process often described as operating independent of task demands and cognitive resources, but emerging evidence suggests it is responsive to a variety of factors (Leow et al., 2018; Kim et al., 2019; Tsay et al., 2021, 2024b; Avraham and Ivry, 2024; Wang et al., 2025). Here, we used clamped feedback to measure implicit adaptation while participants simultaneously performed a visual task. In Exp 1, participants in the DT condition showed a modest increase in implicit adaptation during early learning, even as RSVP performance dropped and partially recovered following the onset of the clamped feedback. In Exp 2, adding response-contingent feedback to the RSVP task (DT_F_) did not prevent the immediate drop in accuracy but facilitated complete recovery of RSVP performance and, more importantly, yielded stronger implicit adaptation relative to the ST condition.

Model-based analyses revealed that these behavioral differences were driven mainly by shifts in error sensitivity. The DT groups showed higher error sensitivity with a seemingly graded response from ST to DT to DT_F_ but modestly lower retention compared with ST. We also observed strong negative correlations between retention and error sensitivity across individuals in the DT conditions, which was weaker to nonexistent in ST. Together, these findings suggest that goal-directed attentional demands not only influence the magnitude of implicit adaptation but also appear to reshape the balance between retention and error sensitivity.

### Tonic arousal as a modulator of error sensitivity

The graded increase in error sensitivity from ST to DT to DT_F_ suggests that goal-directed attention does not interfere with or operate independently of implicit adaptation but may enhance it under certain conditions. We propose this reflects arousal-mediated modulation of error processing in the cerebellar circuits responsible for implicit adaptation. Importantly, this is postulated to be an indirect effect: attentional demands do not modulate implicit adaptation directly but rather induces a tonic arousal state that in turn modulates error sensitivity.

Tonic arousal is a neuromodulatory state that increases neural gain, thereby amplifying the influence of error signals (Aston-Jones and Cohen, 2005; Sara, 2009). Arousal increases with reward, feedback, and attentional demands and is reliably indexed by pupil diameter (Gilzenrat et al., 2010; Eldar et al., 2013; van der Wel and van Steenbergen, 2018). Critically, pupil-linked arousal tracks noradrenergic output from the locus ceruleus (Joshi et al., 2016; Reimer et al., 2016), which modulates cerebellar plasticity at parallel fiber→Purkinje cell synapses where climbing fibers carry error signals (Carey and Regehr, 2009). This provides a plausible neural pathway by which arousal induced by a concurrent goal-directed task could amplify the weights placed on SPEs.

The cerebellar population-coding (CPC) model offers a framework for linking these effects to learning dynamics (Wang and Ivry, 2025). CPC proposes a two-layer network in which cerebellar cortex supports volatile learning—higher error sensitivity, lower retention—while deep cerebellar nuclei support stable learning with the opposite profile. Wang and Ivry (2025) account for various contextual effects through population coding in the input pathway—parallel fibers carrying movement and other contextual information. Although CPC does not incorporate neuromodulation, the present findings suggest a complementary influence: noradrenergic modulation of the plasticity induced by climbing fiber error signals. Such modulation could shift the balance toward volatile processing, amplifying error sensitivity while reducing retention.

Is there evidence to support this account? First, we need to establish that the concurrent task induced differential states of engagement. RSVP accuracy confirms participants performed the task, but the time course differed across conditions. In both DT and DT_F_, accuracy dropped sharply at clamp onset—consistent with attentional capture, whereby attention is automatically allocated to salient sensory features despite their irrelevance to current goals (Theeuwes, 2025). However, the subsequent patterns diverged: DT accuracy only partially recovered during learning, suggesting engagement waned over time, whereas DT_F_ accuracy recovered fully, consistent with response-contingent feedback sustaining engagement.

Second, if engagement regulates arousal and arousal increases error sensitivity, implicit adaptation should track plausible engagement trajectories. Notably, baseline pupil diameter—a proxy for tonic arousal—declines over the course of implicit adaptation (Yokoi and Weiler, 2022), suggesting arousal naturally wanes. This predicts that any boost from concurrent task demands would be transient unless actively sustained. The data are consistent with this prediction: DT showed early enhancement that faded, consistent with engagement waning without RSVP feedback, whereas DT_F_ showed sustained enhancement throughout learning, consistent with RSVP feedback maintaining engagement. Simulations confirm that modeling engagement as a time-varying gain on error sensitivity reproduces the observed group-level trajectories (Extended Data Fig. 6-1).

Third, the parameter structure should match CPC predictions. In the CPC framework, volatile processing is characterized by both higher error sensitivity and lower retention. These are not independent properties but joint consequences of cerebellar cortical dynamics. The single-rate state–space model decomposes behavior into retention and error sensitivity parameters, but it cannot reveal whether these reflect independent mechanisms or a common source. The strong negative correlation between retention and error sensitivity observed under DT conditions suggests the latter: individuals whose arousal most strongly biased the system toward volatile processing showed both higher error sensitivity and lower retention. Together, these findings are consistent with arousal-mediated modulation of cerebellar error processing, though more direct measures of arousal, such as pupillometry, would strengthen this interpretation.

### Automatic, but not autonomous

Recent work has partially clarified the relationship between implicit adaptation and attention. Wang et al. (2025) found that implicit adaptation is insensitive to spatial attention—directing attention toward or away from feedback did not affect learning. Secondary tasks attenuated adaptation under some conditions but only when attended stimuli changed from trial-to-trial, suggesting imperfect generalization between contexts rather than resource competition. This led them to suggest that implicit adaptation is both automatic and resource-independent.

Our findings are compatible with both automaticity and resource independence. First, participants adapted despite explicit instructions to ignore the task-irrelevant feedback, consistent with the obligatory nature of this learning process (Mazzoni and Krakauer, 2006; Morehead et al., 2017). Second, the concurrent task enhanced rather than spared or impaired implicit adaptation, which is the opposite of what resource competition would predict. Moreover, RSVP accuracy and implicit adaptation were unrelated, providing no evidence that the tasks drew on a common resource-limited system.

Our findings also indicate that implicit adaptation was enhanced under conditions of elevated engagement, which is consistent with modulation by tonic arousal. This suggests that while implicit adaptation may not rely on resource-limited systems, like attention or working memory, cognitive states seem to nonetheless influence this form of learning. Such cross-system influence is consistent with the view that neuromodulatory systems operate broadly, amplifying gain across perceptual, cognitive, and motor circuits (Aston-Jones and Cohen, 2005; Mather et al., 2016). Implicit adaptation is automatic but not autonomous: it operates outside voluntary control yet remains open and responsive to the changing physiological states of the individual.

### Alternative explanations

One might ask whether implicit adaptation was suppressed in ST rather than DT and DT_F_ being enhanced. We consider three such accounts.

Spatial suppression arising from selection history is one possibility. Repeatedly ignoring a stimulus at a fixed location can induce spatial suppression that spreads to nearby regions (Theeuwes, 2025). If clamped feedback fell within this suppressive field, its salience and ability to drive adaptation may have been reduced (Theeuwes et al., 2022). However, this account faces several challenges. First, the cursor endpoint may fall outside the suppressive field, though estimates of this field (2–5° visual angle) derive from search displays with different spatial structures (Wang and Theeuwes, 2018). Second, even if some suppression occurred in ST, this cannot explain why DT_F_ produced stronger implicit adaptation than DT as both conditions involved attending to the RSVP stream. Third, spatial suppression does not predict the strong negative correlation between retention and error sensitivity observed under DT conditions, nor the divergent RSVP time courses across DT and DT_F_. These features implicate a mechanism that tracks engagement rather than one that attenuates error signals in ST.

A second possibility is that implicit adaptation and RSVP monitoring compete for shared cognitive resources, with adaptation attenuated once demands exceed available capacity. However Wang et al. (2025) systematically varied cognitive load and found no evidence for resource sharing. Increasing working memory demands (zero-back vs one-back) produced clear differences in task difficulty but no difference in adaptation magnitude. An auditory secondary task also failed to attenuate adaptation. In our data, individual differences in RSVP accuracy were uncorrelated with the magnitude of implicit adaptation. If participants operating near capacity showed reduced adaptation, this relationship should emerge. Finally, the graded pattern (ST < DT < DT_F_) is difficult to reconcile with a capacity account. If RSVP monitoring depletes resources needed for adaptation, DT and DT_F_ should show equivalent (or reversed) effects, not a monotonic increase. Together, these findings suggest implicit adaptation is resource-independent.

A third possibility invokes logic similar to shared-error models (Albert et al., 2022): in standard visuomotor rotation, adding a secondary task could enhance implicit adaptation by impairing explicit strategy use. However, clamped feedback eliminates task error and removes any utility of strategic reaiming. The short, stable RT across learning, suggest that participants were not implementing parametric reaiming strategies. Without an explicit strategy, the shared-error mechanism cannot explain enhanced adaptation under DT conditions.

### Limitations

A central limitation is that we did not directly measure arousal or engagement. Our interpretation rests on the assumption that concurrent task demands elevated tonic arousal, but this remains an indirect inference. Pupillometry would provide a better proxy of arousal state (Joshi et al., 2016), even if it is imperfect (Larsen and Waters, 2018). Similarly, dimensional measures related to arousal reactivity, such as anxiety, may help explain the individual differences. Without such measures, the link between engagement, arousal, and cerebellar modulation remains a plausible but unconfirmed pathway.

While the group sample sizes were modest, they were sufficient to detect moderate effects and are consistent with recent work (Morehead et al., 2017; Kim et al., 2018; Avraham et al., 2021; Tsay et al., 2021, 2024b). We used Bayesian analysis, which provides uncertainty quantification rather than relying on point estimates or binary significance tests (Kruschke, 2014; McElreath, 2018). While the reported effects were not large in absolute terms, the practical relevance of a small effect depends on the context. Failure to correct a small error may be negligible when reaching for a coffee mug but could mean the difference between landing on the green or in a sand trap when golfing.

Finally, we observed less asymptotic adaptation (10–12°) than reported in other studies (15–20°). This is unlikely to reflect antisavings from practice trials, as clamp direction was alternated to prevent cumulative learning before the experiment started. Other factors may have contributed, including the use of a vertical display (Liew et al., 2018) and extended baseline exposure (Avraham and Ivry, 2024).

### Future directions

While group-level patterns are informative, our broader goal is to understand how learning mechanisms operate within individuals. Cognitive traits, such as spatial working memory, appear to influence the balance between implicit and explicit learning (Anguera et al., 2010; Christou et al., 2016; O’Bryan et al., 2025), consistent with theoretical accounts of the cognitive contributions to motor learning (Seidler et al., 2012). Our findings extend this logic from traits to states: goal-directed attention can modulate implicit adaptation and likely does so differently across individuals.

Prior studies have shown that re-exposure to clamped feedback consistently attenuates implicit adaptation (Avraham et al., 2021). As such, we opted for a between-subjects design, but future work should test whether similar patterns are observed within individuals. Importantly, Avraham and Ivry (2024) showed that implicit adaptation can be preserved across multiple exposures if feedback is withheld during washout. These findings support the feasibility of within-subject designs, enabling stronger inferences about individual-level effects.

Tsay et al. (2024a) recently proposed a framework emphasizing how cognitive processes shape explicit, strategic components of motor learning. Our results suggest that cognition also interacts with implicit adaptation, though likely through contextual and neuromodulatory pathways rather than direct resource sharing. A complete account of motor learning will require understanding how cognitive traits and states modulate implicit and explicit systems. Identifying the boundary conditions and contextual factors that shape implicit adaptation remains a compelling direction for future work.

### Conclusion

Implicit adaptation is automatic and appears to be resource-independent—it operates outside voluntary control and does not draw on resource-limited cognitive systems. These properties are often taken to imply that implicit adaptation is insulated from cognitive influences. The present findings challenge this view. Goal-directed attentional demands enhanced implicit adaptation, with a pattern consistent with arousal-mediated modulation of cerebellar error processing. Implicit adaptation is automatic but not autonomous: while it operates outside voluntary control, it remains open to the broader physiological context in which movements are made. Thus, how the nervous system calibrates movement may depend not only on the errors it experiences but also on the states in which it experiences them.

## Synthesis

Reviewing Editor: Nicholas Priebe, The University of Texas at Austin Decisions are customarily a result of the Reviewing Editor and the peer reviewers coming together and discussing their recommendations until a consensus is reached. When revisions are invited, a fact-based synthesis statement explaining their decision and outlining what is needed to prepare a revision will be listed below. The following reviewer(s) agreed to reveal their identity: Eugene Poh.

Two reviewers evaluated the manuscript "Automatic, but not inflexible: Implicit adaptation is modulated by goal-directed attentional demands." The authors describe interactions between attentional demands and implicit adaptation. Both reviewers agreed that the manuscript provides compelling evidence that cognitive load impacts motor adaptation. The reviewers raised a few questions, detailed below, which should strengthen the rigor of the manuscript and clarify the analyses and interpretations of the work.

Major Issues:

1. The description of the RSVP task.

It would be helpful to provide a clearer description of the clamped feedback paradigm, the RSVP task, and the rationale for considering the observed changes in hand path as implicit adaptation-particularly since participants are instructed to ignore the cursor.

It would be helpful to clarify why clamped feedback represents an adaptation task as opposed to more traditional visuomotor rotation or gain adaptation task and to explain what proposed or previously demonstrated sensorimotor error drives adaptation if participants are told to ignore the cursor and told that it is noncongruent feedback, and cite the relevant studies.

In addition, there should be a greater discussion of the acclimation trials and implications. Since participants complete 20 clamped feedback trials before the main experiment, one should consider whether this induces partial adaptation and therefore reflecting savings during the experiment, rather than naïve adaptation, and address implications for interpreting the results. In reviewing other studies of this paradigm and the data presented here, there is a fair amount of learning that actually happens in the first 20 trials.

2. The timecourse of the RSVP performance

In terms of results, additional data visualizations would be valuable, including the time course of RSVP performance, baseline differences across groups, and overlays of model predictions with empirical data. Reporting correlations between RSVP performance and hand-path learning metrics would also help clarify the relationship between cognitive task performance and motor learning.

The choice of cycles 13-17 and 46-50 to define early and late learning appears arbitrary. The authors do not provide a justification for these intervals, and it is unclear whether the effects observed are dependent on these specific windows. Recent work (e.g., Avraham et al., 2025, eNeuro) has advocated for cluster-based, nonparametric permutation tests to identify time windows where group differences are statistically reliable without relying on a priori assumptions. The authors should consider reanalyzing their data using this approach to validate their findings and mitigate any bias introduced by predefined intervals.

3. The inclusion of reaction time and movement time data

The absence of reaction time (RT) and movement time (MT) data is a critical omission, as it limits the interpretability of both the dual-task manipulation and the underlying learning processes. The authors interpret the effects observed under clamped feedback as evidence of implicit learning; however, prior research has raised the possibility that re-aiming strategies may still be engaged even when visual feedback is task-irrelevant. RT/MT data are essential for evaluating this possibility. Specifically, in the context of the RSVP dual-task design, knowing when participants initiated their movements relative to the RSVP stream is crucial for determining whether sensory prediction error (SPE) processing occurred concurrently with attentional demands. Given the brevity of the reaching movement (~5 cm), MTs are likely under 400 ms; if participants routinely waited until after the RSVP stream ended to initiate movement, the tasks would be temporally segmented, and attentional interference during SPE processing would be minimal. Conversely, short RTs would indicate true temporal overlap, lending stronger support to the authors' interpretation that increased attentional engagement modulates implicit adaptation. Without these temporal measures, it remains unclear whether the observed effects reflect modulation of implicit recalibration, strategic processes, or broader context-dependent interactions. The authors should acknowledge these alternative interpretations and report RT and MT distributions to substantiate their claims.

4. Model fits

The model fitting reveals a negative correlation between retention and error sensitivity, but this trade-off is an expected artifact of fitting a single-rate state-space model: increases in one parameter often compensate for decreases in the other. As a result, the model captures the empirical data but does not offer substantial explanatory power beyond what can be observed in the adaptation curves themselves. Fitting a simple exponential function may yield similar interpretability. The authors should better justify the use of the model and clarify what novel insight it provides beyond descriptive summaries. Also, the distributions of retention and error sensitivity (Figure 6A and 6B) appear quite similar across conditions, aside from a few outliers.

5. Describing the Bayesian analysis

The manuscript uses advanced Bayesian metrics (eg. exaggeration ratio) that are not commonly understood. It would be helpful to include a short intuitive explanation of these terms so that readers unfamiliar with Bayesian methods can interpret the results more readily.

Furthermore, the interpretation of the dual-task effects should be more cautious. While the Bayesian statistics support a small enhancement of adaptation, the effect is modest, the lower bound of the credible interval is near zero, only 89% HDIs are reported rather than the more conventional 95%, and alternative explanations-such as competition for explicit resources-should be discussed alongside the proposed tonic arousal account. Similarly, the authors might avoid overemphasizing the additional effect benefit seen in the DTF group (where participants received feedback on performance in the RSVP task), especially given differences between DTF and DT groups lack statistical support. E.g., "Increased attentional engagement further enhanced implicit adaptation"

6. Providing a direct comparison of model and data in figure 6C

Figure 6C would benefit from a direct comparison between the model predictions and empirical data across conditions. For instance, overlaying the model's posterior predictive trajectories alongside the actual group means (as done in Figures 3A and 5A) would help validate model fit and clarify the extent to which the learning curves are explained by the inferred parameters.

7. Interpretating the cognitive influence on motor adaptation

While the authors show a cognitive influence on the change in hand path angle, the reason for the behavioral change might be due to different factors that what is reported in the discussion. In terms of the interpretation, while one possible that tonic arousal induced by RSVP increased implicit adaptation, another possibility is that engagement in the RSVP interfered with the ability of participants to use an explicit strategy to counteract/ignore the clamped feedback that was limiting adaptation during the learning block. The RSVP task may reduce explicit resource availability. This is particularly notable in the fact that differences in performance on the RSVP task seemed to be larger across groups when compared to changes in the motor learning/implicit adaptation task. In sum, it is important to consider whether RSVP-induced competition for cognitive resources, rather than an arousal effect, better explains the observed results.

Minor Issues:

1. In Figures 3B, 3C, 5B, and 5C, lines connecting the individual data points are missing. Including these would improve the interpretability of within-subject changes across conditions.

2. Please define "stimulus-driven attentional capture" for readers not as familiar with this concept.

3. In the methods RT is reference as part of the exclusion criteria. Please clarify if this means reach time (i.e., movement time) or reaction time.

4. Please clarify the directionality of the hand path change in response to the clamped feedback - does it move towards or away from the clamped feedback.

5. Related to Major Issue #3, it would be helpful to include more details about the timing including reaction time and movement time across learning blocks to evaluate for potential changes related to RSVP performance. It seems the participants were instructed to "shoot through" the target, but was there any movement time cutoff or feedback related to too slow of trials?

6. How straight were the path trajectories? Please present some measure (e.g., path length ratio) of path trajectory to confirm participants complied with this instruction and that there weren't a lot of adjustments made in the movements.

7. In the section "RSVP performance was selectively disrupted by clamped feedback", the way ST group performance is described is a bit confusing. The ST group was just responding to the prompt as opposed to completing the RSVP task. Rephrasing this finding to more accurately reflect the meaning of performance in the ST group could improve clarity. As a supplementary/additional figure, it would be helpful to see individual performance on the ST group's RSVP task similar to how it is shown in the DT group (Figure 2).

8. For RSVP performance in Experiment 2, it is recommended to add baseline and learning averages with confidence intervals, consistent with Experiment 1.

9. Related to Major Issue #2, it would be helpful to see a figure of time series of RSVP performance across the experiment. The authors state the absence of performance feedback may have reduced the motivational salience of the RSVP task, making it more difficult to sustain attention over time for experiment 1. It would be helpful for authors to present temporal information of performance throughout the learning block in order to make this point. This would point to a gradual decline in performance on the RSVP task versus a step decline due to the clamped feedback that might require more explicit working memory to counteract it. This could support or refute the claim that RSVP performance declines gradually (or not) and clarify whether effects reflect resource competition and/or mental fatigue.

10. Please compare and comment on baseline differences between groups (in particular DT and DTF groups). The DTF group appears to have worse performance at baseline compared to the DT group (Figure 4, large percentage of people that performed below 60% at baseline). The learning block RSVP percentage between DTF and DT are actually similar (to slightly worse in the DTF group) so the feedback didn't make participants perform better on the RSVP task. Additionally, when looking at individual subject data, many subjects actually show an improvement in the RSVP task during learning for the DTF group. Is it possible that the RSVP task is just a distraction task for the motor learning component?

11. Collapsing across all groups, does RSVP performance correlate with hand angle learning/adaptation across participants.

12. Related to Major Issue #6, plotting of the model simulations and empirical data in the same figure allows for an assessment of model fit visually.

13. Please comment on how error sensitivity and retention in the model are related to RSVP performance.

## Author Response

Synthesis Statement for Author (Required):

Two reviewers evaluated the manuscript "Automatic, but not inflexible: Implicit adaptation is modulated by goal-directed attentional demands." The authors describe interactions between attentional demands and implicit adaptation. Both reviewers agreed that the manuscript provides compelling evidence that cognitive load impacts motor adaptation. The reviewers raised a few questions, detailed below, which should strengthen the rigor of the manuscript and clarify the analyses and interpretations of the work.

Response: Thank you for taking the time to provide such thoughtful and thorough feedback. It revealed a lot of important nuances that were critical to refining and expanding the results, interpretation, and discussion. The revised manuscript is, in our opinion, much improved due to the quality of the reviewer and editorial feedback. We responded to each major and minor issue in turn and did our best to cross-reference responses. In most cases, analyses conducted to address these concerns were either 1) incorporated directly into the revised manuscript or 2) included as extended data.

In this revision, we substantively reframed the manuscript to move beyond a descriptive account of behavioral changes toward a neurophysiologically plausible explanation of how attentional demands modulate implicit adaptation. The revised manuscript now emphasizes that implicit adaptation is "automatic but not autonomous," operating outside conscious control while remaining responsive to the broader neuromodulatory context. To support this account and address the reviewers' concerns, we incorporated comprehensive kinematic analyses of reaction time (RT), movement time (MT), and total action duration (TAD), confirming that reaching movements were straight, ballistic, and executed in parallel with the RSVP task.

Furthermore, we strengthened the computational modeling section by providing a clearer justification for the state-space model and conducting parameter recovery simulations. These simulations demonstrate that the negative correlation observed between retention and error sensitivity in dual-task conditions reflects genuine individual differences rather than artifacts of the model fitting procedure. Additionally, we refined the analysis of the RSVP task by moving from a single learning epoch to a more granular temporal analysis that identifies an immediate "onset drop" following the introduction of the clamped feedback followed by differential recovery patterns across Exp 1 and Exp 2.

To facilitate the review process, we provided a marked-up version of the revised manuscript where substantive changes are indicated with yellow highlights. We made editorial changes to fix typos and reduce excess text wherever possible. The highlighted text indicates modifications made in direct response to reviewer requests, as well as more substantive rewrites or additions.

Major Issues 1. The description of the RSVP task.

It would be helpful to provide a clearer description of the clamped feedback paradigm, the RSVP task, and the rationale for considering the observed changes in hand path as implicit adaptation-particularly since participants are instructed to ignore the cursor.

It would be helpful to clarify why clamped feedback represents an adaptation task as opposed to more traditional visuomotor rotation or gain adaptation task and to explain what proposed or previously demonstrated sensorimotor error drives adaptation if participants are told to ignore the cursor and told that it is noncongruent feedback, and cite the relevant studies.

In addition, there should be a greater discussion of the acclimation trials and implications. Since participants complete 20 clamped feedback trials before the main experiment, one should consider whether this induces partial adaptation and therefore reflecting savings during the experiment, rather than naïve adaptation, and address implications for interpreting the results. In reviewing other studies of this paradigm and the data presented here, there is a fair amount of learning that actually happens in the first 20 trials.

Response: We thank the reviewers for these helpful suggestions. We have clarified both the conceptual and methodological descriptions of the clamped feedback paradigm and expanded our explanation of the practice block.

In the Introduction, we now describe the clamped-feedback task as one in which the feedback cursor is non-contingent: its radial position matches the hand, but its angular position is fixed relative to the target. Participants are informed that the feedback does not reflect their actual movement and are instructed to move directly to the target. Nonetheless, we and others reliably observe gradual, automatic changes in hand angle, consistent with implicit adaptation driven by sensory prediction error (Kim et al., 2018; Morehead et al., 2017). We also clarified that the RSVP task imposed a competing visual signal, providing the basis for our manipulation of attentional demands.

In the Methods, we added a sentence explicitly noting that clamped feedback elicits gradual, automatic changes in hand angle driven by sensory prediction error rather than task error or reward, reflecting implicit adaptation (Kim et al., 2018; Morehead et al., 2017). We also clarified the procedures for the three feedback conditions (veridical, clamped, and no feedback) and the participant instructions.

Regarding the practice block, participants were exposed to clamped feedback for 20 trials, but we omitted a critical detail: the exposure consisted of alternating pairs of counterclockwise (+45{degree sign}) and clockwise (-45{degree sign}) clamps, preventing adaptation from accumulating across trials. This safeguard was implemented based on pilot data showing that sustained exposure to a fixed clamp direction produced measurable implicit adaptation, as the reviewers correctly anticipated.

Finally, we expanded the Discussion to acknowledge potential factors that may explain why the overall magnitude of adaptation (~10-12{degree sign}) was slightly lower than in some prior reports, including the use of a vertical display, which may reduce adaptation compared to horizontal setups, and that the inclusion of dual task practice effectively extends the baseline period, which can attenuate adaptation (Avraham et al., 2024).

2. The timecourse of the RSVP performance a. In terms of results, additional data visualizations would be valuable, including the time course of RSVP performance, baseline differences across groups, and overlays of model predictions with empirical data. Reporting correlations between RSVP performance and hand-path learning metrics would also help clarify the relationship between cognitive task performance and motor learning.

Response to (a): We added time series for ST (Figure 3-1), DT (Figure 3), and DTF (Figure 4), as well as direct comparisons of the dual-task groups (Figure 4-1). We also report correlations between RSVP accuracy and implicit adaptation (see Minor Comment 11) and between RSVP accuracy and learning parameters (see Minor Comment 13). None of these relationships were credible, reinforcing the independence of RSVP performance and implicit adaptation for predicting individual differences. b. The choice of cycles 13-17 and 46-50 to define early and late learning appears arbitrary. The authors do not provide a justification for these intervals, and it is unclear whether the effects observed are dependent on these specific windows. Recent work (e.g., Avraham et al., 2025, eNeuro) has advocated for cluster-based, nonparametric permutation tests to identify time windows where group differences are statistically reliable without relying on a priori assumptions. The authors should consider reanalyzing their data using this approach to validate their findings and mitigate any bias introduced by predefined intervals.

Response to (b): The predefined windows-baseline (cycles 6-10), early learning (cycles 13-17), and late learning (cycles 46-50)-correspond to meaningful phases of adaptation. The baseline window captures performance after task familiarization; early learning begins at cycle 13 to allow the initial response to the clamp to stabilize; late learning captures asymptotic performance. For the RSVP analysis, we additionally examined an onset window (cycles 11-12). This choice was motivated by the time series data, which revealed an abrupt drop in accuracy at clamp onset followed by gradual recovery-a pattern obscured when learning was treated as a single epoch in the original submission.

We acknowledge that window selection involves judgment. To address this, we conducted sensitivity analyses by shifting windows forward (early learning) or backward (late learning). For early learning, effects were stable or slightly larger when shifting the window forward, indicating that cycles 13-17 provided a conservative estimate. For late learning, adjacent windows yielded nearly identical estimates: convergence in Exp 1 (DT-ST ~0.5-0.8{degree sign}, all HDIs including zero) and sustained enhancement in Exp 2 (DTF-ST ~3.3-3.6{degree sign}, with windows 44-48 and 45-49 excluding zero). Thus, our predefined window (cycles 46-50) was again conservative. Full sensitivity results are reported in Figure 5-3.

We also provide a continuous, uncertainty-aware analysis in Figure 5-2, which estimates group differences at each cycle using bootstrap resampling with 89% HDIs. This serves as an estimation-focused alternative to cluster-based permutation tests, which are designed to control family-wise error rates across hypothetical replications-an unnecessarily conservative criterion for single studies where exact replication is unlikely. Moreover, cluster-based approaches require specifying a t-threshold, which introduces its own arbitrariness. Our approach prioritizes effect estimation and uncertainty quantification over binary significance testing.

3. The inclusion of reaction time and movement time data The absence of reaction time (RT) and movement time (MT) data is a critical omission, as it limits the interpretability of both the dual-task manipulation and the underlying learning processes. The authors interpret the effects observed under clamped feedback as evidence of implicit learning; however, prior research has raised the possibility that re-aiming strategies may still be engaged even when visual feedback is task-irrelevant. RT/MT data are essential for evaluating this possibility. Specifically, in the context of the RSVP dual-task design, knowing when participants initiated their movements relative to the RSVP stream is crucial for determining whether sensory prediction error (SPE) processing occurred concurrently with attentional demands. Given the brevity of the reaching movement (~5 cm), MTs are likely under 400 ms; if participants routinely waited until after the RSVP stream ended to initiate movement, the tasks would be temporally segmented, and attentional interference during SPE processing would be minimal. Conversely, short RTs would indicate true temporal overlap, lending stronger support to the authors' interpretation that increased attentional engagement modulates implicit adaptation. Without these temporal measures, it remains unclear whether the observed effects reflect modulation of implicit recalibration, strategic processes, or broader context-dependent interactions. The authors should acknowledge these alternative interpretations and report RT and MT distributions to substantiate their claims.

Response: We appreciate this feedback and agree that temporal measures are needed for evaluating whether reaching overlapped with the RSVP task and determining whether participants may have relied on explicit strategies. We regret not including these data.

To start, we define reaction time (RT) and movement time (MT). RT is the duration from trial start to movement initiation, which was measured via a velocity threshold. We also previously examined a position threshold, such as when the cursor exits the starting ring, and observed no meaningful differences. MT is defined as the duration from movement onset until the cursor exceeds the target distance, which was measured via a position threshold. The units for these variables are milliseconds. We added details to the Methods to explain that these variables were computed and examined as part of the analysis. Moreover, we added a hard cap of 500 ms for MT to exclude slow movements (<10 cm/s), which is noted in the Methods and had a minor impact on excluded trials.

We added RT, MT, and TAD analyses to the Results and Figure 5-1. RT was short (~440 ms across groups) and stable across learning, with no evidence of systematic drift. These latencies are consistent with implicit adaptation, particularly given the absence of strict RT constraints, and are not suggestive of deliberate re-aiming. MTs were also brief (~185 ms) and relatively invariant over learning, indicating that movements were not prolonged to permit online corrections. Critically, TAD was well below the 1500 ms trial duration (~630 ms across groups), confirming that the reaching and RSVP tasks overlapped temporally.

To test whether group-specific differences improved model fits, we conducted leave-one-out cross-validation. For all three measures (RT, MT, TAD), models with group-specific parameters did not improve predictive accuracy (e.g., RT: ΔELPD = -0.5, SE = 1.3), supporting the conclusion that groups did not differ in reach timing. Together, these analyses confirm that participants initiated and executed movements rapidly, in parallel with RSVP processing, making it unlikely that the observed effects can be explained by re-aiming strategies or temporal segmentation of the tasks.

4. Model fits The model fitting reveals a negative correlation between retention and error sensitivity, but this trade-off is an expected artifact of fitting a single-rate state-space model: increases in one parameter often compensate for decreases in the other. As a result, the model captures the empirical data but does not offer substantial explanatory power beyond what can be observed in the adaptation curves themselves. Fitting a simple exponential function may yield similar interpretability. The authors should better justify the use of the model and clarify what novel insight it provides beyond descriptive summaries. Also, the distributions of retention and error sensitivity (Figure 6A and 6B) appear quite similar across conditions, aside from a few outliers.

Response: The reviewers raise important questions about model justification. We address these by first clarifying the scientific motivation for computational modeling, then describing how we ensured our findings reflect genuine individual differences.

Why model at all? The learning curves in Figure 6c look similar across conditions-and that is precisely why modeling is necessary. Similar behavioral outcomes can arise from different underlying computations. In simple state-space models, implicit adaptation reflects the aggregate output of two processes: error-based learning and time-dependent forgetting. Behavioral measures do not dissociate these contributions, while the state-space model can.

This dissociation matters because attentional demands could, in principle, affect error sensitivity, retention, or both. The modeling revealed a specific pattern: error sensitivity increases systematically from ST to DT to DTF, while retention shows only modest decreases. An exponential fit may capture the curves equally well but would be purely descriptive. That is, it cannot distinguish whether a given asymptote reflects strong learning offset by forgetting, or weaker learning with better retention. As we clarify in the Results, the model addresses a specific scientific question: similar behavioral outcomes can arise from different underlying computations, and the model distinguishes them.

Ruling out fitting artifacts: The reviewer correctly notes that retention and error sensitivity can trade off during fitting: for any single participant, parameter combinations producing similar steady-states create a ridge in the likelihood surface. This is an issue of within-subject identifiability. It does not, however, imply that between-subject correlations are artifactual. Such an artifact would require all participants to reach similar steady-states, effectively sampling along a shared ridge. Our participants showed substantial heterogeneity in asymptotic adaptation (1{degree sign} to 27{degree sign}), which breaks this degeneracy.

We tested this directly via parameter recovery. We sampled retention and error sensitivity independently from the fitted group-level distributions (expected r = 0), simulated learning trajectories with measurement noise from our sample, and refit the model. Across 1,000 simulated experiments per condition, we compared recovered correlations to those observed in our data (Figure 7-1). The fitting procedure introduced a modest negative bias (median r ≈ -0.14 to -0.23). For ST, the observed correlation (r = -0.29) was compatible with this artifact. For DT and DTF, the observed correlations (r = -0.81, -0.73) exceeded nearly all recovered values. Thus, we are confident that the tradeoff in dual-task conditions reflects genuine individual differences.

On the distributions and apparent outliers: The parameter shifts across conditions are modest, and yet these modest shifts are sufficient to reproduce the behavioral patterns in Figure 6c. Note that we added the cycle-level data as requested. This is what good model fits yield: small changes in parameters can yield measurable behavioral differences.

The points flagged by boxplot conventions are not pathological. Retention and error sensitivity are bounded on (0,1). Distributions pushed against bounds necessarily produce skewed tails. MATLAB's default 1.5×IQR whiskers assume unbounded normal distributions and mischaracterize observations from bounded distributions as outliers. Our Bayesian approach also addresses this concern directly: we apply a multivariate Student's t likelihood to logit-transformed parameters, respecting the bounded parameter space while estimating degrees of freedom from the data to accommodate extreme observations.

5. Describing the Bayesian analysis The manuscript uses advanced Bayesian metrics (e.g. exaggeration ratio) that are not commonly understood. It would be helpful to include a short intuitive explanation of these terms so that readers unfamiliar with Bayesian methods can interpret the results more readily. Furthermore, the interpretation of the dual-task effects should be more cautious. While the Bayesian statistics support a small enhancement of adaptation, the effect is modest, the lower bound of the credible interval is near zero, only 89% HDIs are reported rather than the more conventional 95%, and alternative explanations-such as competition for explicit resources-should be discussed alongside the proposed tonic arousal account. Similarly, the authors might avoid overemphasizing the additional effect benefit seen in the DTF group (where participants received feedback on performance in the RSVP task), especially given differences between DTF and DT groups lack statistical support. E.g., "Increased attentional engagement further enhanced implicit adaptation" Response: Agreed. Please request additional clarification as needed. We are happy to make these changes. The goal is not to hide behind obscure statistical methods. The Bayesian methods are much more intuitive in our opinion, even if unfamiliar. For instance, when we report a mean is 20 [10,30], we can make intuitive probabilistic statements such as: given the model and data, the probability that the mean is between 10 and 30 with X%. This is not possible with frequentist methods. We added a short paragraph to the Methods to clarify Bayesian measures used throughout the manuscript. For example, we added probability statements to help the reader interpret certain effects (i.e., Pr(DT > ST).

Regarding the modest effect sizes: we agree and have been more careful throughout to characterize uncertainty rather than make binary claims. HDIs contain the most credible values. Even if a distribution crosses a threshold, like zero, that doesn't tell us how plausible that value is relative to the mode or some other value. Regardless, we revised the section header for Exp 2 to avoid overstating the DTF-DT contrast, which was smaller and more uncertain than the DTF-ST comparison.

Note that the 89% HDI is a principled choice in Bayesian analysis (McElreath, 2018), not a strategy to narrow intervals. The 89% HDI excludes the 11% of posterior values with the lowest credibility. Because inclusion is determined by credibility rather than arbitrary convention, this approach guards against both overstating effects (by excluding implausibly large values) and understating them (by excluding implausibly small values). In contrast, a 95% frequentist confidence interval has no such interpretation. It is a fixed-width region across hypothetical replications, independent of how plausible any value is.

6. Providing a direct comparison of model and data in figure 6c Figure 6c would benefit from a direct comparison between the model predictions and empirical data across conditions. For instance, overlaying the model's posterior predictive trajectories alongside the actual group means (as done in Figures 3A and 5A) would help validate model fit and clarify the extent to which the learning curves are explained by the inferred parameters.

Response: Agreed. We added the empirical cycle means (dots) to Figure 6c alongside the posterior predictive simulations (lines and shading). This allows direct comparison of model predictions and observed data. We updated the figure caption to clarify that dots represent empirical cycle means, solid lines represent the posterior predictive median, and shaded regions represent the 89% HDI.

7. Interpretating the cognitive influence on motor adaptation While the authors show a cognitive influence on the change in hand path angle, the reason for the behavioral change might be due to different factors that what is reported in the discussion. In terms of the interpretation, while one possible that tonic arousal induced by RSVP increased implicit adaptation, another possibility is that engagement in the RSVP interfered with the ability of participants to use an explicit strategy to counteract/ignore the clamped feedback that was limiting adaptation during the learning block. The RSVP task may reduce explicit resource availability. This is particularly notable in the fact that differences in performance on the RSVP task seemed to be larger across groups when compared to changes in the motor learning/implicit adaptation task. In sum, it is important to consider whether RSVP-induced competition for cognitive resources, rather than an arousal effect, better explains the observed results.

Response: We appreciate this concern and have expanded the Alternative explanations section of the Discussion to address it more thoroughly.

The reviewer's hypothesis invokes logic similar to shared-error models (Albert et al., 2022). RSVP engagement could reduce cognitive resources available for explicit strategy, thereby "releasing" implicit adaptation. We evaluated this possibility with several lines of evidence.

First, we examined RT, MT, and total action duration (TAD = RT + MT). If RSVP reduced resources for strategic re-aiming, RT should be longest in ST (where resources are fully available for strategy). However, RTs were short (~430-450 ms), stable across learning, and did not differ between groups. These latencies are much shorter than those typically observed for strategic re-aiming to a 45{degree sign} perturbation without time constraints (~1100 ms; McDougle &Taylor, 2019), and movement timing did not predict adaptation differences.

Second, and more fundamentally, the shared-error mechanism requires an explicit component to impair. Clamped feedback eliminates task error and removes any basis for strategic re-aiming. The aiming location is the target. The cursor follows a fixed path regardless of hand position. Participants were aware that re-aiming was not effective and were instructed to move directly to the target. Without an explicit strategy operating, there is nothing for RSVP to "release." This is the main appeal of the clamped feedback paradigm.

We also addressed two related accounts in the expanded Discussion: spatial suppression arising from selection history, and general resource competition between adaptation and RSVP monitoring. Each is plausible at first glance but fails to explain the full pattern of results-particularly the graded enhancement (ST < DT < DTF), the temporal dynamics of the RSVP task, and the A-b correlation structure under dual-task conditions.

Minor Issues 1. In Figures 3B, 3C, 5B, and 5C, lines connecting the individual data points are missing. Including these would improve the interpretability of within-subject changes across conditions.

Response: We appreciate the suggestion. However, the study employed a between-subjects design, so the plotted points reflect distinct participants rather than repeated measures. This is stated in the Methods, and therefore connecting lines would not be appropriate.

2. Please define "stimulus-driven attentional capture" for readers not as familiar with this concept.

Response: Thank you for pointing this out. We removed this from the Introduction for clarity and define it where it is brought it. Briefly, attentional selection often distinguishes between goal-driven (top-down) and stimulus-driven (bottom-up) processes (Theeuwes, 2010). Goal-driven processes are guided by intentions and goals (e.g., searching for your keys), whereas stimulus-driven processes reflect the involuntary pull of salient environmental features (e.g., a high-contrast distractor attracting gaze).

3. In the methods RT is reference as part of the exclusion criteria. Please clarify if this means reach time (i.e., movement time) or reaction time.

Response: Another good catch. This clarification was addressed in our response to Major Issue #3 and the associated revisions. Nothing to add here.

4. Please clarify the directionality of the hand path change in response to the clamped feedback - does it move towards or away from the clamped feedback.

Response: This was not stated in the Results. However, in the Methods we noted, 'Positive hand angle values denote clockwise deviation, such that positive changes in hand angle were expected during exposure to clamped feedback.' To clarify: hand angle shifted away from the clamped feedback, consistent with implicit adaptation-participants adapted as if trying to counteract the perceived error. We added a brief clarification to the Results to reinforce this point.

5. Related to Major Issue #3, it would be helpful to include more details about the timing including reaction time and movement time across learning blocks to evaluate for potential changes related to RSVP performance. It seems the participants were instructed to "shoot through" the target, but was there any movement time cutoff or feedback related to too slow of trials? Response: We agree and have added a confirmatory check section to the Results to address Major Issue #3. This section reports reaction time (RT), movement time (MT), and total action duration (TAD = RT + MT), summarized in Figure 5-1. Although we did not conduct exhaustive correlation analyses, these measures showed no systematic change following the introduction of clamped feedback or across learning, suggesting that variations in RT or MT are unlikely to account for the observed effects.

We did not impose real-time MT cutoffs or provide speed feedback (e.g., "move faster"). However, to address this concern, we implemented an additional outlier criterion flagging trials with MT > 500 ms. This removed 43 trials (0.29 %) that were evenly distributed across time. While this threshold is intentionally conservative to maximize data retention, note that mean MT was < 200 ms and ~98.6 % of trials had MT < 300 ms. Thus, even without feedback, participants consistently followed the instruction to "shoot through" the target.

6. How straight were the path trajectories? Please present some measure (e.g., path length ratio) of path trajectory to confirm participants complied with this instruction and that there weren't a lot of adjustments made in the movements.

Response: We agree and examined movement straightness to confirm that participants executed smooth, ballistic reaches as instructed. Straightness ratio was quantified as the ratio of the straight-line distance to the hand path length. As shown below, the distribution of straightness ratios was tightly clustered near 1 (mean = 0.9923, median = 0.9959, min = 0.6683, max = 0.9999). A beta distribution provided a close fit to the data (red line). The shaded region indicates the 89 % HDI. Nearly all trials were straight-line reaches, with 98.6 % > 0.95 and 99.6 % > 0.90, confirming that participants complied with the instruction to "shoot through" the target and made minimal online corrections.

7. In the section "RSVP performance was selectively disrupted by clamped feedback", the way ST group performance is described is a bit confusing. The ST group was just responding to the prompt as opposed to completing the RSVP task. Rephrasing this finding to more accurately reflect the meaning of performance in the ST group could improve clarity. As a supplementary/additional figure, it would be helpful to see individual performance on the ST group's RSVP task similar to how it is shown in the DT group (Figure 2).

Response: We agree with this feedback. We clarified the description of ST group performance in the Results section of Exp 1 to make it clear that participants responded to a simple prompt after each reach, rather than performing the RSVP task during reaching. We also added Figure 3-1, which visualizes ST performance. This change hopefully improves clarity and transparency while keeping the Results section focused.

8. For RSVP performance in Experiment 2, it is recommended to add baseline and learning averages with confidence intervals, consistent with Experiment 1.

Response: Thanks for pointing this out and apologies for the inconsistency. This information was added to the Exp 2 results.

9. Related to Major Issue #2, it would be helpful to see a figure of time series of RSVP performance across the experiment. The authors state the absence of performance feedback may have reduced the motivational salience of the RSVP task, making it more difficult to sustain attention over time for experiment 1. It would be helpful for authors to present temporal information of performance throughout the learning block in order to make this point. This would point to a gradual decline in performance on the RSVP task versus a step decline due to the clamped feedback that might require more explicit working memory to counteract it. This could support or refute the claim that RSVP performance declines gradually (or not) and clarify whether effects reflect resource competition and/or mental fatigue.

Response: We added the time series for ST (Figure 3-1), DT (Figure 3), and DTF (Figure 4), as well as a direct comparison of the dual-task groups (Figure 4-1). As indicated in our response to major comment 2, the time series reveal a step decline at clamp onset followed by gradual recovery, not a gradual decline throughout learning. The abrupt, time-locked disruption suggests attentional capture by the salient error clamp.

The incomplete recovery in DT raised the possibility that engagement declined over time in the absence of RSVP feedback. This was the motivation for Exp 2, independent of the drop in response to clamp onset. By adding response-contingent feedback, we aimed to sustain engagement with the RSVP task. The complete recovery in DTF supports this interpretation: feedback maintained engagement, which in turn sustained the enhancement of implicit adaptation.

10. Please compare and comment on baseline differences between groups (in particular DT and DTF groups). The DTF group appears to have worse performance at baseline compared to the DT group (Figure 4, large percentage of people that performed below 60% at baseline). The learning block RSVP percentage between DTF and DT are actually similar (to slightly worse in the DTF group) so the feedback didn't make participants perform better on the RSVP task. Additionally, when looking at individual subject data, many subjects actually show an improvement in the RSVP task during learning for the DTF group. Is it possible that the RSVP task is just a distraction task for the motor learning component? Response: We thank the reviewers for raising this concern. In response, we now report epoch-by-epoch RSVP accuracy and provide direct group comparisons between the dual-task groups (Figure 4-1). See response to major comment 2 for the justification to examine onset, early, and late learning epochs relative to baseline.

The reviewers correctly note that DTF appeared to have lower baseline accuracy. But this difference was about 3%. The posterior probability that DT exceeded DTF at baseline was 79%-modest evidence of a difference, though not definitive. This difference persisted through onset (89%) and early learning (83%). However, by late learning, the pattern reversed: DTF had a 61% probability of exceeding DT. We cannot know why baseline accuracy differed between groups. We could note that the credible intervals include zero at each epoch, but that would be inconsistent with how we apply these methods elsewhere. Our working assumption is that the baseline difference reflects sampling variation. The RSVP task is difficult, with accuracy averaging around 67%, and people vary considerably.

As for why some DTF participants appeared to "improve" during learning: this reflects recovery from the onset drop, not improvement above baseline. Figure 4b shows that by late learning, roughly half of participants were above their baseline accuracy and half below-consistent with stable mean performance and individual variability, not systematic improvement.

The feedback manipulation was not intended to boost accuracy above DT levels. It was intended to prevent the offset from baseline observed in DT. DTF returned to baseline by late learning while DT did not. In the original manuscript, we treated learning as a single epoch, which was inappropriate. The transient drop in accuracy was consistently observed across individuals in both groups and was unrelated to implicit adaptation, suggesting that the clamped feedback drew attention away from the RSVP task despite instructions to ignore it. This shows how salient clamped feedback is from an attentional standpoint as we have not observed this systematic drop in RSVP performance with abrupt or gradual visuomotor rotations (Im et al., 2015, 2016, 2022).

Regarding whether the RSVP task is "just a distraction": accuracy was well above chance throughout both experiments, indicating participants performed the task. The onset-specific disruption indicates that the salient error signal captured attention, shifting it away from the RSVP stream. If the RSVP stream served only as irrelevant noise, we would not expect a selective disruption tied to clamp onset, nor differential recovery based on feedback condition. Finally, individual differences in RSVP accuracy were unrelated to implicit adaptation, ruling out a resource tradeoff. Importantly, while individual differences did not predict adaptation, the group-level enhancement was reliable. This dissociation suggests the concurrent task context modulates adaptation through a mechanism other than direct resource competition.

11. Collapsing across all groups, does RSVP performance correlate with hand angle learning/adaptation across participants.

Response: We examined the relationship between RSVP accuracy and implicit adaptation both within groups and collapsed across the dual-task groups (DT and DTF). In Exp 1, correlations between RSVP accuracy changes and adaptation were weak and not credible at any time point (onset, early, late). The same pattern was observed in Exp 2. Collapsing across dual-task groups did not alter these results. Thus, we found no evidence that individual differences in RSVP performance predicted the magnitude of implicit adaptation. We now report both analyses in the Results.

12. Related to Major Issue #6, plotting of the model simulations and empirical data in the same figure allows for an assessment of model fit visually.

Response: This was addressed in our response to Major Issue # 6.

13. Please comment on how error sensitivity and retention in the model are related to RSVP performance.

Response: We examined whether RSVP accuracy predicted the learning parameters using Beta regression with a logit link, given that both the learning parameters and RSVP accuracy are bounded proportions. Pooling across DT and DTF, neither baseline nor late RSVP accuracy was associated with retention (baseline: β = 0.07 [-1.08, 1.22]; late: β = -0.44 [-1.41, 0.54]) or error sensitivity (baseline: β = -0.22 [-1.32, 0.90]; late: β = 0.36 [-0.57, 1.27]). This is consistent with the absence of individual-level correlations between RSVP accuracy and implicit adaptation reported in the behavioral analyses. We report this in the modeling section (without statistics).
