# Supplementary figures and images for: Automatic, But Not Autonomous: Implicit Adaptation Is Modulated by Goal-Directed Attentional Demands
Source: eNeuro. 2026 Mar 13;13(3):ENEURO.0243-25.2026. doi: 10.1523/ENEURO.0243-25.2026 (PMC13002316; doi:10.1523/ENEURO.0243-25.2026)

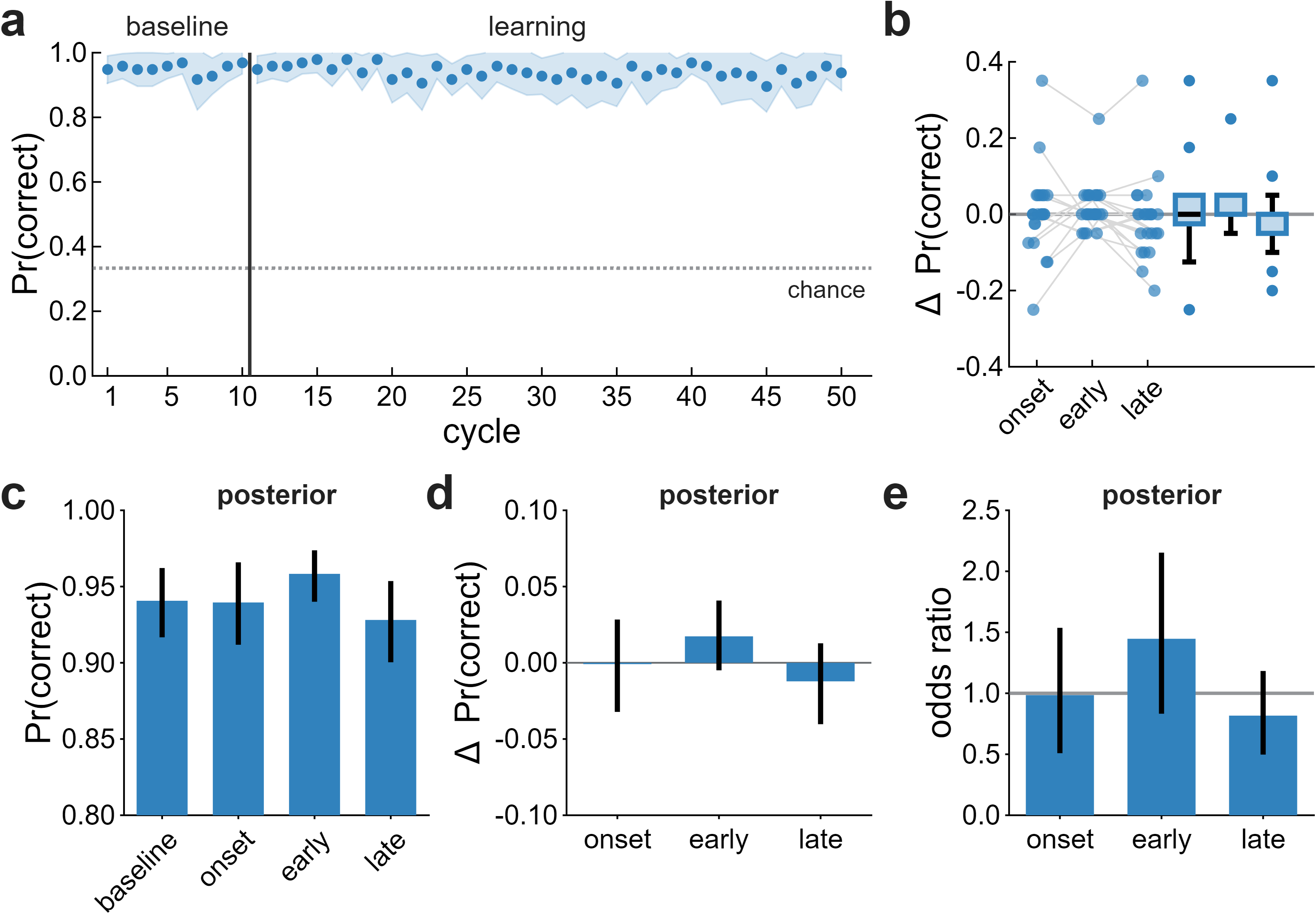

Supplement: Figure 3-1 — Single-task (ST) performance in Exp 1. (a) Proportion of correct responses across baseline and learning. Dots indicate group means; shaded regions indicate ± 2 SEM. The dotted line is chance performance (0.33). Accuracy remained well above chance throughout the experiment. (b) Individual changes in accuracy relative to baseline for each learning epoch: onset (cycles 11–12), early (cycles 13–17), and late (cycles 46–50). (c–e) Posterior summaries from a hierarchical binomial model showing (c) median accuracy, (d) changes from baseline, and (e) odds ratios relative to baseline. Error bars are 89% HDIs. Changes were centered near zero, indicating that performance was not systematically affected by the clamped feedback. Download Figure 3-1, TIF file. [file eneuro-13-ENEURO.0243-25.2026-s002.tif]

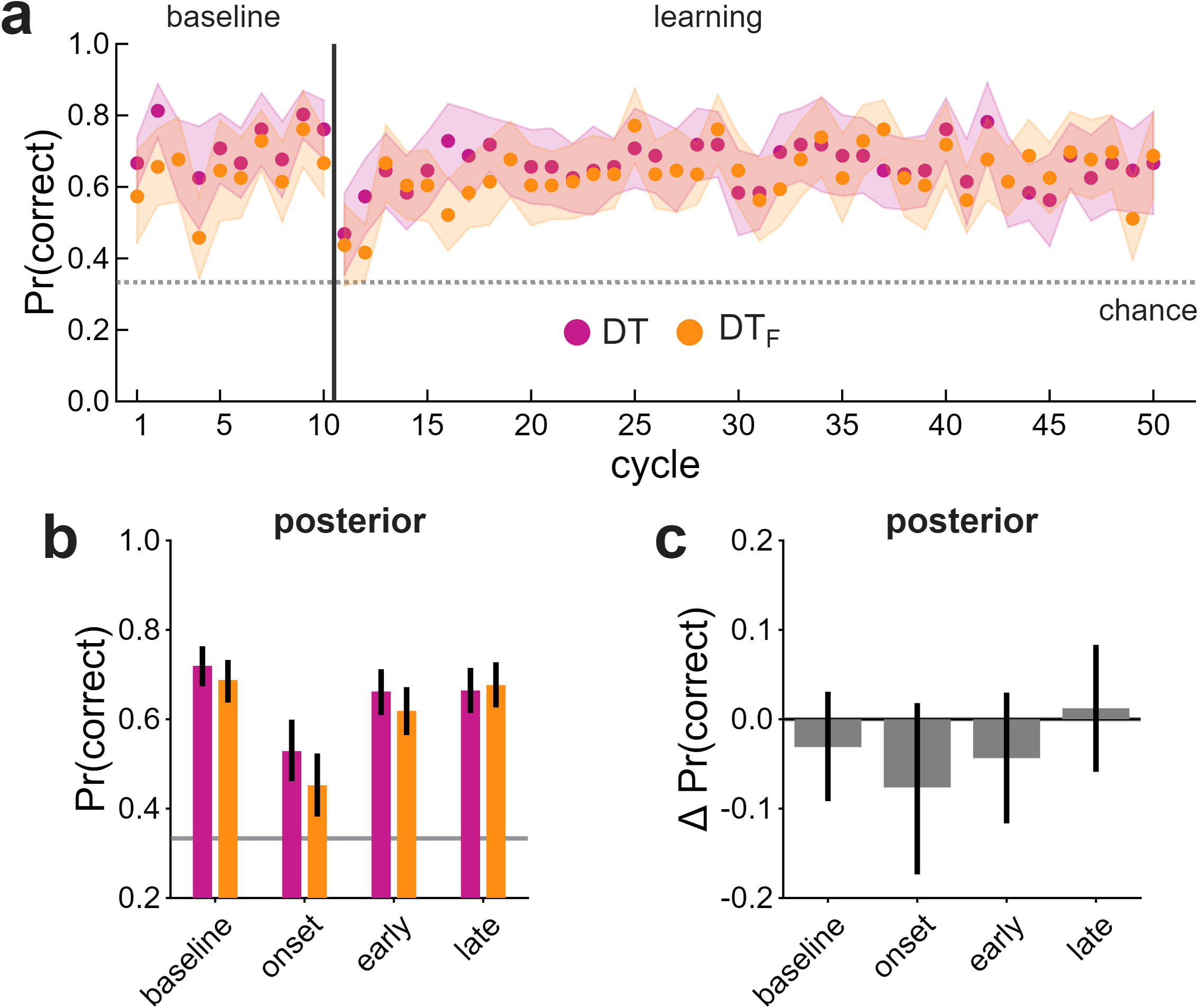

Supplement: Figure 4-1 — Comparison of dual-task performance (DT and DTF). (a) Proportion of correct responses across baseline and learning. Dots indicate group means; shaded regions represent ± 2 SEM. The dotted line indicates chance performance (0.33). (b) Posterior median accuracy with 89% HDIs for each group and epoch. (c) Posterior median group differences (DTF – DT) with 89% HDIs. Group differences were small and spanned zero at all epochs. Download Figure 4-1, TIF file. [file eneuro-13-ENEURO.0243-25.2026-s003.tif]

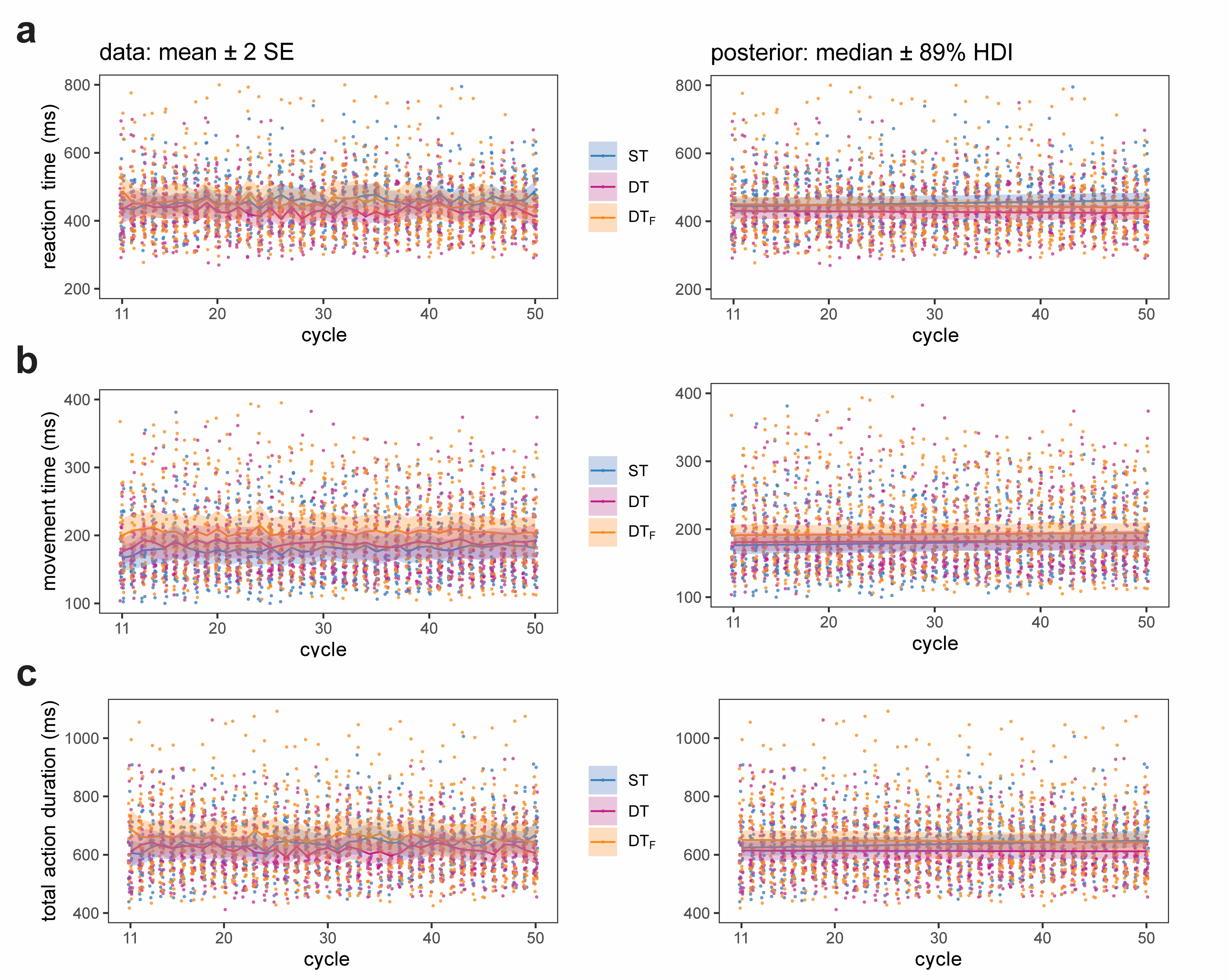

Supplement: Figure 5-1 — Reaction time (RT), movement time (MT), and total action duration (TAD). Each dot represents an individual participant during learning (cycles 11-50). Left panels: data with group means ±2 SEM. Right panels: posterior medians and 89% HDIs. (a) RT was short (440 ms [428, 454]) and stable across learning, with no systematic group differences. (b) MT was also short (184 ms [175, 192]), with no evidence of change over learning, nor any group differences. (c) TAD was consistently less than 1500 ms (629 ms [612, 648]), indicating that the reaching and visual task were executed in parallel. Download Figure 5-1, TIF file. [file eneuro-13-ENEURO.0243-25.2026-s004.tif]

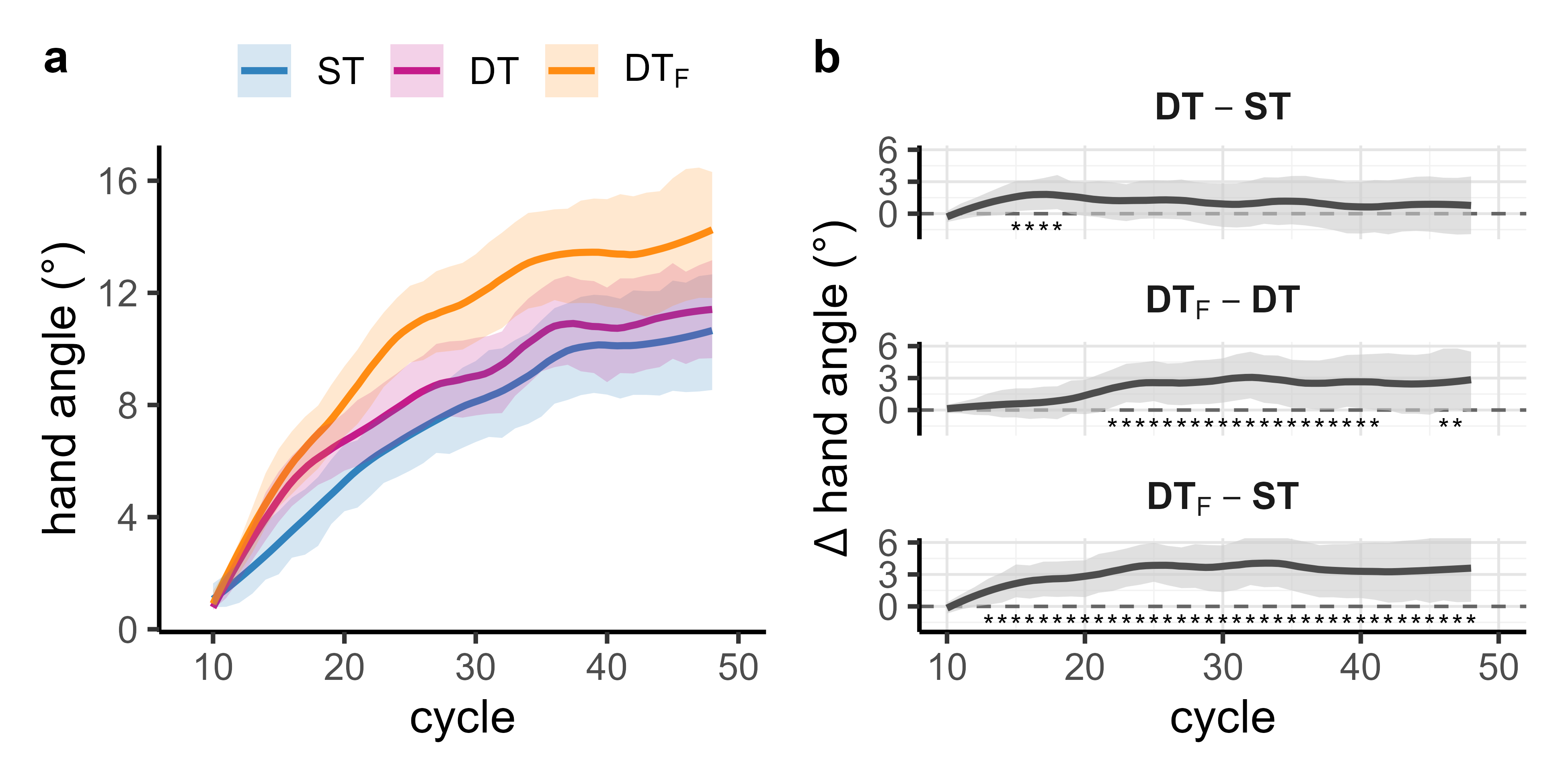

Supplement: Figure 5-2 — Bootstrap estimation of group differences in implicit adaptation. (a) Mean hand angle across cycles for ST, DT, and DTF. Shaded regions denote 89% bootstrap HDIs. (b) Pairwise differences (Δ hand angle) between groups across cycles, with shaded regions indicating 89% HDIs. To reduce cycle-to-cycle noise, participant-level data were smoothed with a five-cycle centered moving average before resampling. Group means and pairwise differences were estimated via bootstrap resampling (10,000 iterations), and 89% HDIs were computed for each cycle. We interpret differences as credible when the 89% HDI for Δ hand angle excluded 0° (asterisks), particularly across contiguous cycles, reflecting a sustained rather than transient divergence. This provides a continuous, uncertainty-based estimate of when and by how much groups differed, serving as an estimation-based alternative to cluster-based permutation tests. The latter are designed to control long-run family-wise error rates across hypothetical replications and are therefore conservative for estimation-focused analyses where an exact replication is rarely conducted. Download Figure 5-2, TIF file. [file eneuro-13-ENEURO.0243-25.2026-s005.tif]

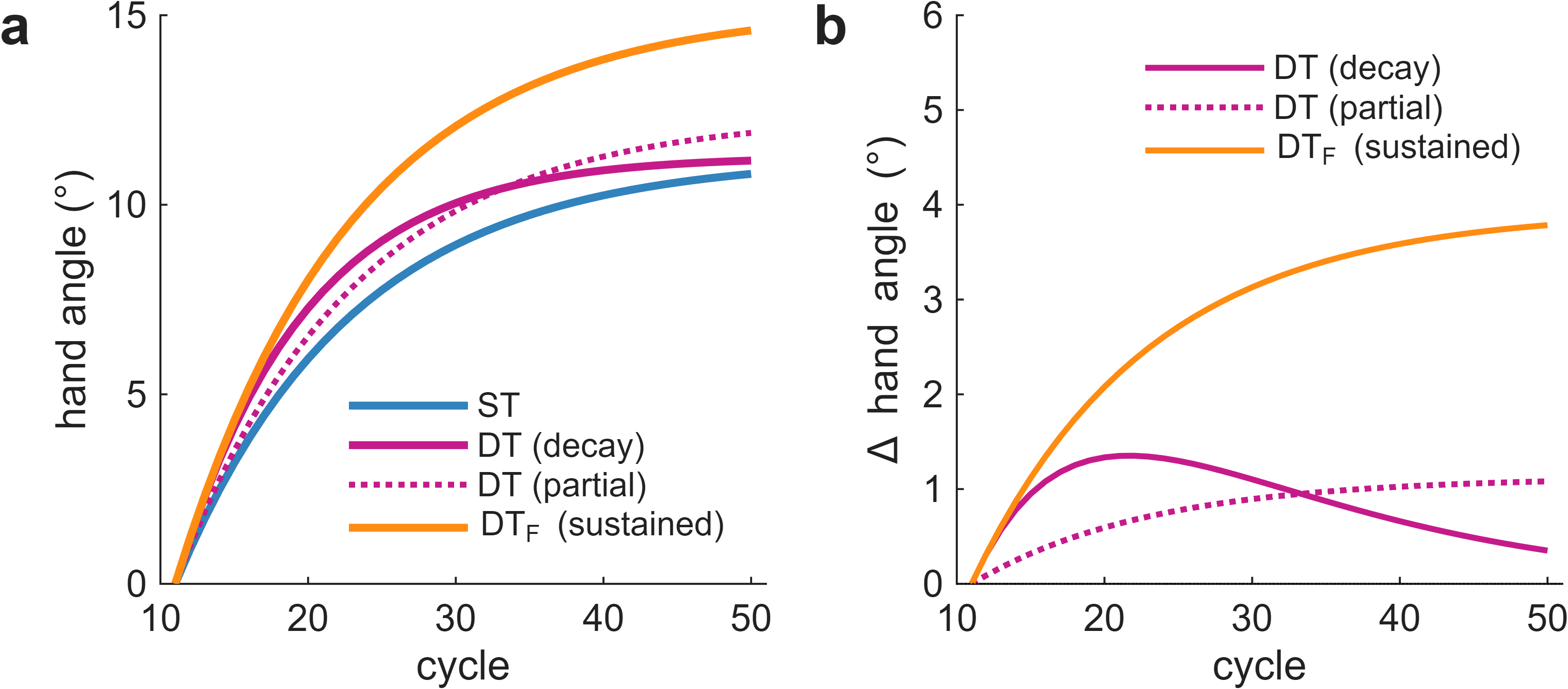

Supplement: Figure 6-1 — Learning trajectories under engagement-modulated error sensitivity. (a) Simulated hand angle across cycles for ST, DT, and DTF. All simulations used identical retention (A = 0.92) and baseline error sensitivity (b = 0.02); conditions differed only in an engagement multiplier, m(n), that scaled error sensitivity on each cycle. For ST, m = 1 throughout. Two DT scenarios are shown: an initial boost that decays back to baseline (solid magenta; m(n) = 1 + 0.35·0.9ⁿ) and a partial but sustained boost (dotted magenta; m = 1.10). For DTF, engagement was elevated and sustained throughout (orange; m = 1.35). (b) Predicted differences from ST. The decaying engagement scenario for DT produces transient enhancement followed by convergence, matching the observed pattern. Any sustained elevation would predict persistent separation from ST, inconsistent with Exp 1. Sustained engagement in DTF produces persistent enhancement, as observed in Exp 2. Download Figure 6-1, TIF file. [file eneuro-13-ENEURO.0243-25.2026-s007.tif]
