# Supplementary material for: Automatic, But Not Autonomous: Implicit Adaptation Is Modulated by Goal-Directed Attentional Demands
Source: eNeuro. 2026 Mar 13;13(3):ENEURO.0243-25.2026. doi: 10.1523/ENEURO.0243-25.2026 (PMC13002316; doi:10.1523/ENEURO.0243-25.2026)
Supplement: Figure 5-3 — Sensitivity analysis for predefined analysis windows. Adjacent five-cycle windows were examined to assess whether group differences depended on window selection. For early learning, windows were shifted forward; for late learning, windows were shifted backward. Asterisks (*) indicate the predefined windows reported in the main text. Results were stable across windows, confirming that the predefined windows provided conservative estimates. Download Figure 5-3, DOCX file. [file eneuro-13-ENEURO.0243-25.2026-s006.docx]

| **Epoch** | **Contrast** | **Window** | **Δ Hand angle (°)** | **89% HDI** | **Effect size (d)** | **89% HDI** |
| --- | --- | --- | --- | --- | --- | --- |
| Early | DT–ST | 13–17* | 1.5 | [0.0, 3.0] | 0.50 | [0.01, 0.98] |
|  |  | 14–18 | 1.7 | [0.1, 3.3] | 0.53 | [0.03, 1.03] |
|  |  | 15–19 | 1.8 | [0.2, 3.4] | 0.53 | [0.03, 1.03] |
|  |  | 16–20 | 1.8 | [0.2, 3.7] | 0.51 | [0.03, 1.03] |
| Early | DT_F_–ST | 13–17* | 2.4 | [0.7, 3.9] | 0.72 | [0.22, 1.25] |
|  |  | 14–18 | 2.4 | [0.8, 4.1] | 0.69 | [0.18, 1.19] |
|  |  | 15–19 | 2.7 | [1.0, 4.6] | 0.73 | [0.21, 1.24] |
|  |  | 16–20 | 2.7 | [0.9, 4.6] | 0.73 | [0.22, 1.22] |
| Late | DT–ST | 46–50* | 0.5 | [–2.6, 3.8] | 0.08 | [–0.39, 0.56] |
|  |  | 45–49 | 0.6 | [–2.5, 3.6] | 0.09 | [–0.37, 0.56] |
|  |  | 44–48 | 0.6 | [–2.4, 3.7] | 0.10 | [–0.36, 0.57] |
|  |  | 43–47 | 0.8 | [–2.2, 3.9] | 0.13 | [–0.33, 0.61] |
| Late | DT_F_–ST | 46–50* | 3.3 | [–0.2, 6.8] | 0.44 | [–0.02, 0.93] |
|  |  | 45–49 | 3.5 | [0.0, 7.0] | 0.46 | [0.00, 0.93] |
|  |  | 44–48 | 3.6 | [0.2, 7.2] | 0.48 | [0.01, 0.94] |
|  |  | 43–47 | 3.3 | [–0.2, 6.7] | 0.45 | [–0.03, 0.92] |
