# Supplementary material for: Automatic, But Not Autonomous: Implicit Adaptation Is Modulated by Goal-Directed Attentional Demands
Source: eNeuro. 2026 Mar 13;13(3):ENEURO.0243-25.2026. doi: 10.1523/ENEURO.0243-25.2026 (PMC13002316; doi:10.1523/ENEURO.0243-25.2026)
Supplement: Figure 7-1 — Parameter recovery simulation table. A potential concern is that negative correlations between retention (A) and error sensitivity (b) can arise as an artifact of model fitting, since different parameter combinations can produce similar steady-state learning. To rule this out, we sampled A and b independently from the group-level posterior distributions (breaking any correlation; expected r = 0), simulated 1,000 learning trajectories per group with measurement noise drawn from the observed residual distributions, and refit the model. We then computed Pearson correlations on the recovered parameter estimates. Simulated r reflects independent sampling (expected r = 0). Recovered r reflects bias introduced by model fitting (median [89% HDI] across simulated experiments). Observed r is the Pearson correlation from participant-level point estimates. Pr(observed ≤ recovered) indicates the proportion of recovered correlations at least as extreme as observed. The fitting procedure introduced modest negative bias. For ST, the observed correlation was compatible with fitting artifact. For DT and DTF, the observed correlations exceeded nearly all recovered correlations, indicating genuine individual differences rather than model degeneracy. Download Figure 7-1, DOCX file. [file eneuro-13-ENEURO.0243-25.2026-s008.docx]

| **Group** | **Simulated *r*** | **Recovered *r*** | **Observed *r*** | **Pr(observed ≤ recovered)** |
| --- | --- | --- | --- | --- |
| ST | 0.00 [-0.34, 0.33] | -0.23 [-0.69, 0.14] | -0.29 | 0.44 |
| DT | 0.00 [-0.37, 0.30] | -0.14 [-0.51, 0.25] | -0.81 | < 0.01 |
| DT_F_ | 0.00 [-0.33, 0.31] | -0.15 [-0.55, 0.17] | -0.73 | < 0.01 |
